# Dynamics of the Heat Stress Response of Ceramides with Different Fatty-Acyl Chain Lengths in Baker’s Yeast

**DOI:** 10.1371/journal.pcbi.1004373

**Published:** 2015-08-04

**Authors:** Po-Wei Chen, Luis L. Fonseca, Yusuf A. Hannun, Eberhard O. Voit

**Affiliations:** 1 Wallace H. Coulter Department of Biomedical Engineering, Georgia Institute of Technology, Atlanta, Georgia, United States of America; 2 The George W. Woodruff School of Mechanical Engineering, Georgia Institute of Technology, Atlanta, Georgia, United States of America; 3 The Cancer Center at Stony Brook Medicine, Stony Brook University, Health Science Center, Stony Brook, New York, United States of America; ETH Zurich, SWITZERLAND

## Abstract

The article demonstrates that computational modeling has the capacity to convert metabolic snapshots, taken sequentially over time, into a description of cellular, dynamic strategies. The specific application is a detailed analysis of a set of actions with which *Saccharomyces cerevisiae* responds to heat stress. Using time dependent metabolic concentration data, we use a combination of mathematical modeling, reverse engineering, and optimization to infer dynamic changes in enzyme activities within the sphingolipid pathway. The details of the sphingolipid responses to heat stress are important, because they guide some of the longer-term alterations in gene expression, with which the cells adapt to the increased temperature. The analysis indicates that all enzyme activities in the system are affected and that the shapes of the time trends in activities depend on the fatty-acyl CoA chain lengths of the different ceramide species in the system.

## Author Summary

The heat stress response in yeast is a model system for elucidating how cells organize and execute complex tasks. While a genomic response to heat is necessary, it is by itself too slow for immediate means of protecting the cell against damage. However, one observes changes in the physiology of the cells within a few minutes, which raises the question of how these changes are facilitated. The present study highlights one aspect of this fast response to heat stress. It deciphers the strategies with which the baker’s yeast *Saccharomyces cerevisiae* changes the concentrations of certain sphingolipids, which subsequently lead to targeted alterations in gene expression. The study is based on *de novo* time series measurements of sphingolipid concentrations, which are analyzed with a complex combination of dynamic modeling, reverse engineering, and optimization.

## Introduction

Decades of research on sphingolipids have documented the enormous importance of this class of lipids in mediating a variety of critical cell functions. Sphingolipids exist in eukaryotic cells, where they serve not only as constituents of membranes but also as second messengers in different signaling transduction pathways. These can trigger specific changes in gene expression in organisms like baker’s yeast and aid the control of cell proliferation, differentiation, cell trafficking and apoptosis in mammals [1–4]. Different sphingolipids often mediate overlapping but distinct cell functions, and it is frequently the balance between different sphingolipid species that evokes a critical response. In particular, the balance among ceramide, sphingosine, and sphingosine-1-phosphate is critical for regulating stress responses, programmed cell death, cell proliferation, differentiation, and cancer survival [5].

The biosynthesis of sphingolipids has been characterized quite well in yeast as well as mammalian cells. It is governed by a complex and highly regulated network of pathways that synthesize and degrade the various sphingolipids and incorporate them into membranes or retrieve them from membranes as the situation demands [6,7]. The complexity of sphingolipid metabolism renders it close to impossible to understand intuitively how sphingolipid-mediated responses to different environmental stresses are launched and coordinated. It is clear that any type of genomic response would require at least 15 to 20 minutes, if not hours, for transcription, translation, and protein activation, depending on the particular species in question. However, the first aspects of many stress responses are observed within a few minutes, if not seconds. This important fact leaves no doubt that some important regulatory mechanisms must be activated immediately upon the onset of stress, and it has been shown that sphingolipids and trehalose belong to the first responders and react to stresses very quickly [8–12]. The sphingolipid-based stress response strategy is highly dynamic and difficult to measure experimentally, but we recently showed that it can be inferred computationally from metabolic profile data in the form of time series, if they are supported by a mathematical model that is analyzed with a customized optimization strategy. In the case of heat stress in *Saccharomyces cerevisiae*, the sphingolipid response seems to be a consequence of temperature-induced conformational changes in essentially all enzymes of the pathway system. The resultant alterations in enzyme activities lead to changes in the metabolic profile of sphingolipids, which secondarily induce the expression of specific genes that are beneficial for an effective heat stress response.

Interestingly, the inferred enzymatic profiles throughout the 30-minute heat stress response show distinct differences, but are similar for enzymes that are grouped into functional modules within the larger pathway system [13]. For example, the key enzymes ceramide synthase, sphingoid base kinase, and hydroxylase, which are all directly involved in sphingolipid biosynthesis and internal material distribution, show similar dynamics. These functional modules imply an intricate coordination of enzyme activities within the heat stress response. Our earlier computational analysis also revealed a rapid initial uptake of substrate and increased sphingolipid biosynthesis, which however lasts only for a few minutes. Afterwards, the key enzymes of *de novo* biosynthesis exhibit decreased activity or stop catalyzing altogether. By contrast, the enzyme Isc1 (IPCase) which is involved in converting inositol phosphorylceramide (IPC) compounds to ceramide, exhibits increased, persistent activation, suggesting that a few minutes after the heat stress begins, sphingolipid materials are retrieved from the membranes and redistributed to serve the cell’s needs. Finally, our computational results showed that after 30 minutes of heat stress, the metabolic profile has essentially returned to its initial state under optimal, cooler temperature, while many enzyme activities are still substantially altered. This observation suggests that the cells assure that the sphingolipid metabolites, as important regulators of cell function, are maintained in, or returned to, a particular metabolic state, which apparently is optimal in some sense, while the corresponding enzyme activities seem to be a means to this end and in themselves may be rather different under optimal and stress conditions.

Recent experimental studies have demonstrated that not only the main sphingolipid metabolites in yeast differentially respond to heat stress, but that even variants of the same key sphingolipids, which differ in the lengths of their lipid backbones and fatty acyl head groups, can play distinct roles in cell signaling [14,15]. These alternate signaling roles of sphingolipids have been receiving increasing attention from academia and the biomedical industry. They also lead to the question of how exactly these different sphingolipid species are synthesized and how cells regulate and control the concentration of each sphingolipid variant.

Again, an experimental investigation of the control strategies used by the cells seems difficult, and we therefore develop computational methods here that allow us to shed light on these strategies. Using the results from our previous analysis quasi as boundary conditions, we narrow our focus on the smaller system of ceramides and design a much more detailed model that accounts for different ceramide species. As in earlier studies, we use Biochemical Systems Theory (BST) as the modeling framework and design a combinatorial modeling approach that includes data preprocessing, dynamic flux estimation, and a modified multiple shooting optimization method. This custom-tailored method allows us to infer changes in enzymatic activities that lead to observed responses of the various ceramide species following heat stress.

### Pertinent Details of Sphingolipid Metabolism

Ceramides form a class of sphingolipids with a lipid backbone and a fatty acyl head group. Distinct variants of ceramides result from different backbones and fatty acyl CoAs. For example, C16-dihydroceramide (C16-DHC) is a ceramide with a DHS backbone and a C16 fatty acyl CoA (palmitoyl CoA) head group. Recent research has revealed that DHC species with long fatty acyl chain lengths, such as C18-DHC, as opposed to very long chain based DHC species, such as C26-DHC, have distinct signaling roles [15,16]. To understand and explain these subtle differences, it is necessary to characterize the metabolic mechanisms of ceramide biosynthesis and utilization.

**Fig 1:**
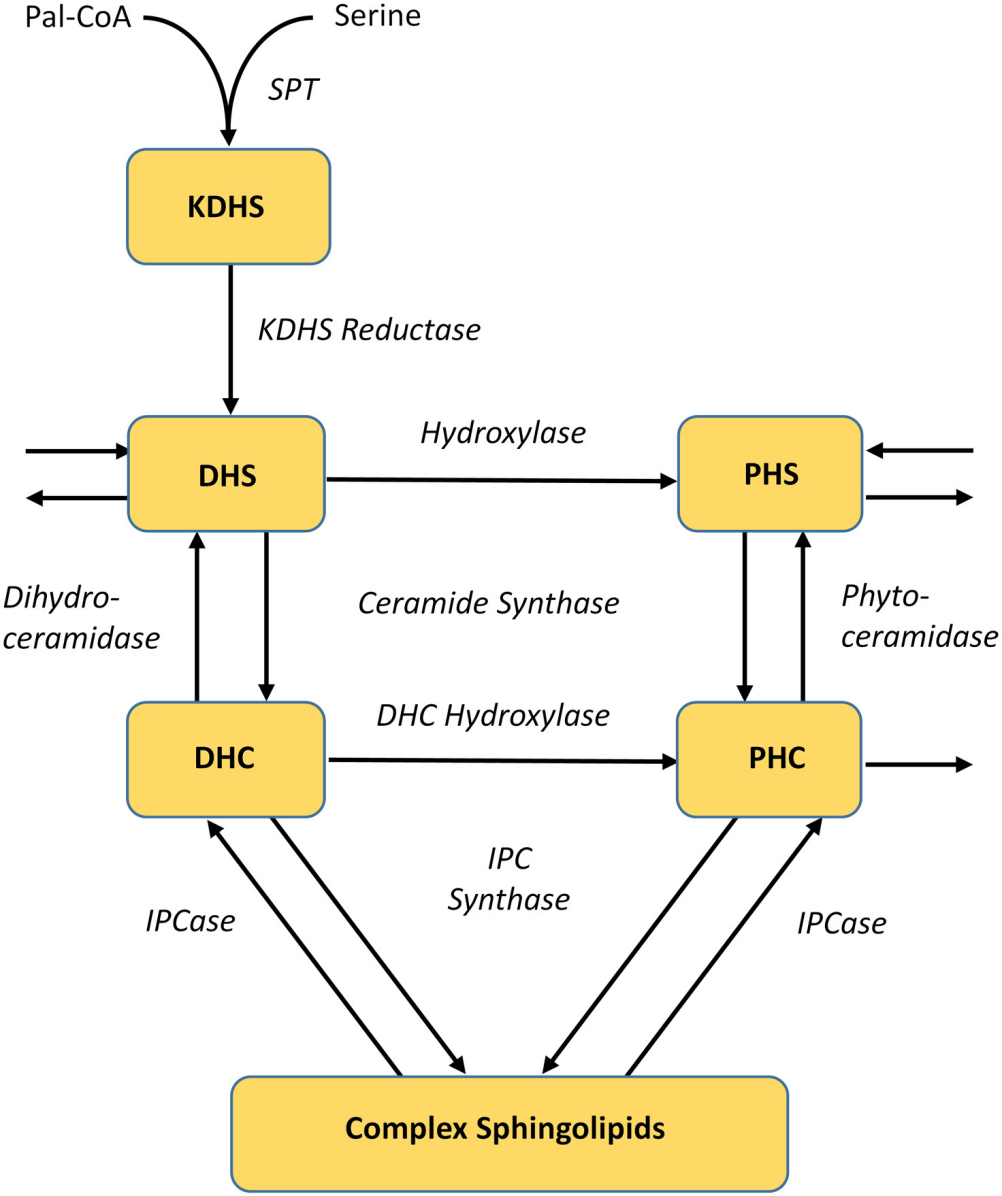
Greatly simplified diagram of ceramide metabolism. Key metabolites are shown in boxes; while enzymes are represented in italics. See Text for abbreviations.

Ceramide can be generated via two paths, namely *de novo* biosynthesis and conversion of inositol phosphoceramide (IPC). *De novo* biosynthesis of sphingolipids is initiated by the condensation of serine and palmitoyl CoA, a reaction which is catalyzed by serine palmitoyltransferase (SPT) (Fig 1). The product, 3-keto-dihydrosphingosine (3KDHS) is quickly reduced by KDHS reductase to dihydrosphingosine (DHS). DHS is a main source of ceramide backbone compounds. It can be converted into different dihydroceramides (DHC), due to multiple options for fatty acyl CoAs, which can serve as substrates for ceramide synthase [17,18]. The reverse reaction, from DHC to DHS, is catalyzed by dihydroceramidase [19]. DHC and DHS are key branch points in the sphingolipid biosynthesis pathway, because hydroxylases [20] can irreversibly convert these compounds into phytoceramide (PHC) and phytosphingosine (PHS), respectively. PHC and PHS may undergo reversible reactions catalyzed by ceramide synthase (PHS to PHC) and phyotoceramidase (PHC to PHS); the forward reaction (PHS → PHC) requires one from among several different fatty acyl CoAs as substrate[8].

*De novo* biosynthesis is not the only option for making sphingolipids available when needed. Equivalent to sphingomyelin in mammalian cells, yeast complex sphingolipids, including inositol phosphoryl-ceramides (IPCs), refer to a class of ceramides with one or two inositol groups attached. They can be formed from DHC and PHC substrates via catalysis by the enzyme IPC synthase. IPC can be irreversibly converted into mannose inositol phosphorylceramide (MIPC), which can furthermore form mannose di-inositol phosphorylceramide (MIP_2_C). Importantly, IPC, MIPC and MIP_2_C all can serve as sources of DHC and PHC, through catalysis by the enzyme IPCase (Isc1). Thus, utilization of IPC compounds serves as the second path of ceramide production [21].

The reactions described in the previous paragraphs form a complex metabolic network, in which different enzyme systems exert subtle control over the proper concentration profile of the various sphingolipid species. Focusing on the ceramide species, namely, DHC and PHC, we can describe the biosynthesis and utilization of DHC and PHC as a cooperation among five enzyme systems consisting of ceramide synthases, ceramidases, IPC synthases, IPCases, and DHC hydroxylases. Ceramide synthases and IPCases mediate the formation of DHC and PHC, while ceramidases and IPC synthases use DHC and PHC as substrates. The hydroxylation reaction, catalyzed by DHC hydroxylases, converts DHC into PHC.

Our previous results [13] indicate that the enzymatic activities within the sphingolipid pathway can be grouped by their responses to heat stress, and thereby suggest that the various sphingolipid species are components of highly coordinated enzyme modules. An important detail we discovered is that the *de novo* biosynthesis of sphingolipids is activated immediately upon heat stress, but sustained only for a few minutes. This phase is followed by the activation of IPC utilization, which leads to the accumulation of sphingolipids that are retrieved from complex sphingolipids in the membranes. In this earlier, larger model, a single variable represented the concentration of all dihydroceramides (DHCs), irrespective of their fatty-acyl chain lengths, and a second variable represented the collective concentration of all phyotoceramides (PHCs). The DHC and PHC levels are controlled by five enzyme classes, namely ceramide synthase, dihydroceramidase and phyotoceramidase, IPC synthase, IPCase (Isc1), and dihydroceramide hydroxylase. A simplified representation is shown in Fig 1; for details of the sphingolipid pathway at large, see [13,22,23]. Ceramide synthase and DHC hydroxylase showed a similar pattern of activity changes, with a strong initial uptake of material followed by a weak profile toward the end. By contrast, dihydro- and phytoceramidase showed entirely different patterns. While dihydroceramidase exhibited very strong activation in the first 10 minutes, which then receded, phytoceramidase exhibited less pronounced peaks of activation. IPC synthase and IPCase had similar activities, again beginning with a sharp spike, which decreased much slower than for ceramide synthase.

While these inferred activity patterns shed some light on how cells regulate their sphingolipid or ceramide contents, they are too coarse to account for changes in the regulatory patterns that govern distinct ceramide species. The present study fills this gap.

## Results

The enzyme activities inferred computationally in our previous study [13] demonstrate how yeast resets its ceramide concentrations under heat stress. The alterations in activities are globally coordinated and grouped, and show distinct regulatory patterns across different enzymes and even ceramides with different fatty acid chains. In the following, we first briefly summarize pertinent findings from this previous research, which focused on heat-stress induced enzyme responses within the broader sphingolipid pathway system, and then zero in on details of the responses among enzymes directly associated with ceramides of different fatty acyl chain lengths.

### Alterations in Enzymatic Activities within the Ceramide Pathway System

**Fig 2:**
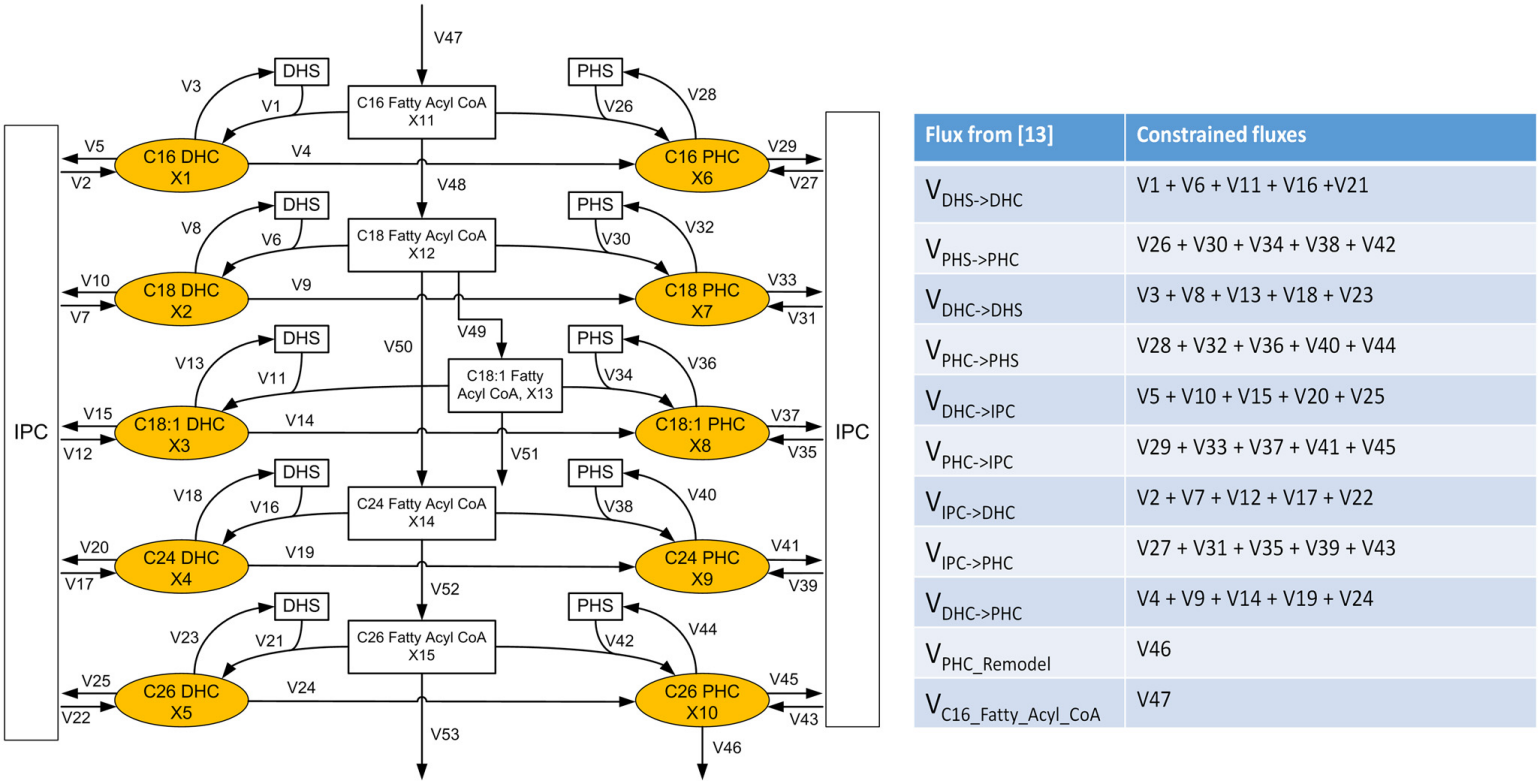
Structure of the proposed model of ceramide dynamics. Left panel: Detailed pathway of ceramide biosynthesis and utilization. The pathway consists of three major subsystems: synthesis and utilization of DHC (left column), synthesis and utilization of PHC (right column), and fatty acid elongation and desaturation (center column). V1, V6, V11, V16, V21, V26, V30, V34, V38 and V42 represent fluxes catalyzed by ceramide synthase. V3, V8, V13, V18, V23, and V28, V32, V36, V40 and V44 represent fluxes catalyzed by dihydroceramidase and phytoceramidase, respectively. Reactions exiting the system to the left or right, namely V5, V10, V15, V20, V25, V29, V33, V37, V41 and V45, represent fluxes catalyzed by IPC synthase, while reactions entering the system from the left or right, V2, V7, V12, V17, V22, V27, V31, V35, V39 and V43, represent fluxes catalyzed by IPCase (Isc1). V4, V9, V14, V19, V24 represent DHC hydroxylase. The vertically shown reactions represent reactions catalyzed by other enzymes, such as remodelase (V46), fatty acid elongases (V47, V48, V50, V51, V52, and V53), and desaturase (V49). The boxes marked “IPC” summarily account for the complex sphingolipids IPC, MIPC, and M(IP)_2_C and their inter-conversions. This simplification seems reasonable as all three, IPC, MIPC and M(IP)_2_C, can serve as sources for the production of DHC and PHC. Right panel: The dynamic flux estimation is partly based on some fluxes whose magnitudes we took from our previous model [13]. The table summarizes these fluxes and indicates on the right how they constrain fluxes in our present model.

As mentioned above, the dynamics of ceramide species is governed by five groups of enzymes, which are shown in Fig 1 along with SPT and KDHS, which catalyze prior reactions steps. Our overall strategy for zeroing in on this sub-pathway is similar to our previous approach, although the detailed account here requires several methodological modifications, which are detailed in the section *Materials and Methods*. As an illustrative example, consider dihydroceramidase. In our previous study, this enzyme catalyzed a single reaction step from DHC to DHS. Now, dihydroceramidase is involved in five reactions, from C14 and C16 DHC to DHS, C18 DHC to DHS, and so on, to the conversion of C26 and C26:1 DHC into DHS. The relationships between fluxes in the previous and in the present model are shown in the right panel of Fig 2. References regarding the enzymatic reactions can be found in the section *Pertinent Details of Sphingolipid Metabolism*. The refined ceramide model discussed here shares many reactions with the previous sphingolipid model, but is expanded toward details regarding the availability of specific substrates.

The reactions of this detailed subsystem are presented in the left panel of Fig 2. The left side of the diagram shows the different DHC species, categorized by chain lengths, and the right side the corresponding PHC species. The processes in the center are associated with elongation and desaturation. We designed a dynamic pathway model of this system with methods of Biochemical Systems Theory [24–27], which have been widely documented in the literature.

Using the computational approach described in the *Materials and Methods* Section, we set out to infer detailed changes in enzymatic activities from time series measurements that had been generated specifically for the purpose of better understanding the heat stress response [14]. The data demonstrate that during the 30-minute heat stress response, different ceramide species respond in dramatically different ways, with some accumulating, others being reduced over time, and yet others maintaining a relatively stable concentration. We used these data to parameterize the model, while allowing the enzyme activity levels to change every three minutes. Custom-tailored methods, reminiscent of multiple-shooting optimization, were used for this purpose.

**Fig 3:**
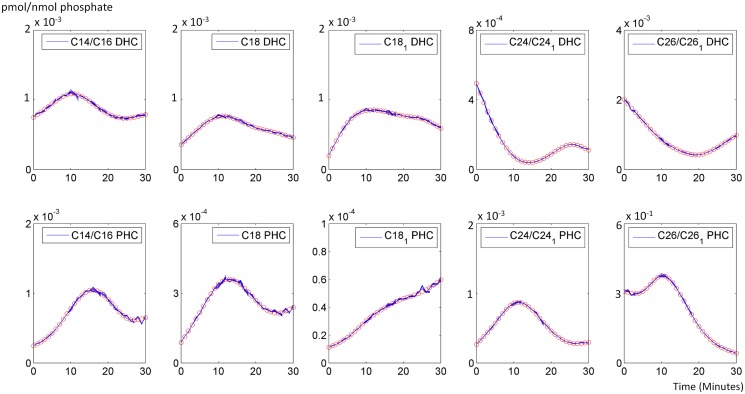
Smoothed and interpolated concentrations of ceramide species. The concentrations were reconstructed with the model, using inferred enzymatic activities; for raw data, see Material and Methods section. The X-axis represents the 30 minutes of heat stress, while the Y-axis represents the concentrations of pertinent ceramides. In each graph, red circles represent the smoothed and interpolated trends in experimental data, and the segmented blue lines represent the corresponding ceramide concentrations computed with the dynamic model using optimized enzymatic activities. The discontinuities are due to the optimization method, which was gleaned from multiple shooting methods. See Materials and Methods Section and Text for further details.

As a first diagnostics of the parameterization, we tested the goodness of fit of the model with time varying enzyme activities for the smoothed, interpolated ceramide data, which are given in Fig 3; the raw data are shown in the *Materials and Methods* Section. Specifically, every three minutes the enzyme activities in the model were allowed to change, and this procedure yielded a reasonably good fit. The results are not entirely smooth due to the abrupt changes in enzyme activities between modeling windows. Even shorter windows were not considered necessary, considering the magnitude of noise in the data and our intent merely to characterize trends in enzyme activities, whereas longer windows (5 or 10 minutes) did not lead to satisfactory fits. As a consequence, the comparatively minor changes in sphingolipids do not seem to affect their levels much. Therefore, much smaller fluctuations in these major metabolites during stress environment should be expected in order for cells to minimize side effects in other pathways.

The model with these optimized parameter values fits the smoothed, interpolated metabolite data quite well, and large-scale simulations (see Materials and Methods) confirmed that the model settings are robust to moderate perturbations. Our computational inferences of enzyme activities, derived from these metabolic measurements, reveal the dynamic patterns that generate the various ceramide species with different carbon chain lengths.

One should note that the shapes, rather than the magnitudes, of the inferred fold changes are more indicative of actual changes. The reason is that, due to insufficient experimental information, our method only infers the product of a rate constant and the corresponding enzyme activity, but cannot assign numerical values to the two factors separately. This situation is analogous to determining *V*_*max*_ in a Michaelis-Menten reaction, rather than *k*_*cat*_ and the total enzyme concentration.

#### 1. Ceramide synthase

**Fig 4:**
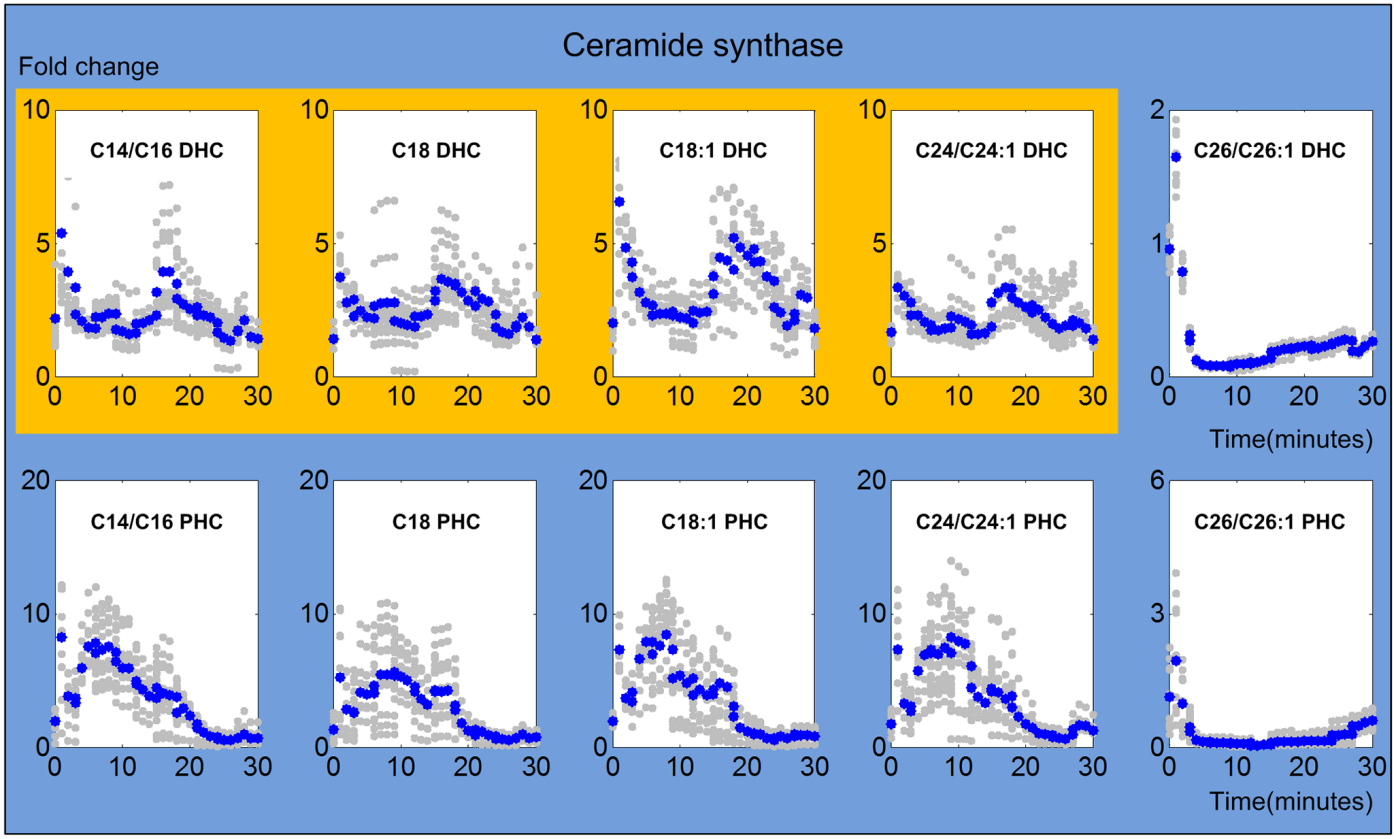
Trends in ceramide synthase activities. The upper panel shows ceramide synthase activities toward DHC, while the lower panel shows ceramide synthase activities toward PHC. The X-axis represents the 30 minutes of heat stress and the Y-axis represents fold changes in activities. In each plot, the blue and gray dots represent averaged and individual enzyme activities, respectively. The yellow and green shading marks similar enzyme activity patterns involved in DHC (top panel) and PHC (bottom panel) synthesis, respectively. It is unknown whether the concentrations of fatty acyl CoAs remain constant during the experiment. If they change dynamically, the shown trends refer to the combined changes in fatty acyl CoAs and ceramide synthase activities.

Ceramide synthases are involved in ten model reactions that are associated with the synthesis of C14/C16, C18, C18:1, C24/C24:1 and C26/C26:1 DHC and PHC. Their dynamically changing activities are shown in Fig 4. All ceramide synthases are activated immediately when heat stress commences, which is presumably due to the Arrhenius effect, but this spike in activity only lasts for two or three minutes. This observation is directly consistent with our previously published results [13], as well as other experimental findings [10,28]. Furthermore, the dynamic patterns of ceramide synthases show a trend based on different fatty acyl CoAs that are used as substrates. While the activities of ceramide synthases that use C14/C16 more or less return to the baseline after a few minutes, it appears that the activities exhibit a modest second peak between 15 and 20 minutes, which however, could be an artifact. In strong contrast, the activity for C26 undershoots after the initial spike and only recovers toward the end of the heat stress experiment. It is known that there is only one ceramide synthase associated with three genes (LAC1, LAG1 and LIP1) in yeast, which shows remarkably different substrate preferences [18,29,30].

The activities of ceramide synthase toward the synthesis of PHCs show different trends (shaded green in Fig 4). For substrates between C14 and 24, the activities gradually increase for about six to eight minutes and subsequently return slowly to their original values, which they reach after about 25 minutes. Interestingly, the activity for the very long chain substrates C26 and C26:1 is very similar for the synthesis of C26 DHC: it spikes to about 4- or 5-fold and immediately drops back to the baseline.

A note is in order. The estimated ceramide synthase activities are actually products of ceramide synthase activities and the concentrations of the corresponding fatty acyl CoAs. Unfortunately the literature offers very little information about the concentrations of fatty acyl CoAs in yeast during short periods of heat stress. If it is legitimate to assume that the concentrations of fatty acyl CoAs remain close to their steady state values during the 30 minutes of heat stress that we study here, then the estimated trends can directly be interpreted as time-dependent changes in enzyme activities. Similarly, if the fatty acyl CoA concentrations exhibit a step change during heat stress, the baseline activities of ceramide synthase would be affected, but the dynamic trends would again directly refer to enzyme activities. However, if the fatty acyl CoA concentrations exhibit dynamical changes during the 30 minutes of heat stress, then the estimated trends are to be interpreted as the products of fatty acyl CoA concentrations and ceramide synthase activities.

#### 2. Ceramidase

**Fig 5:**
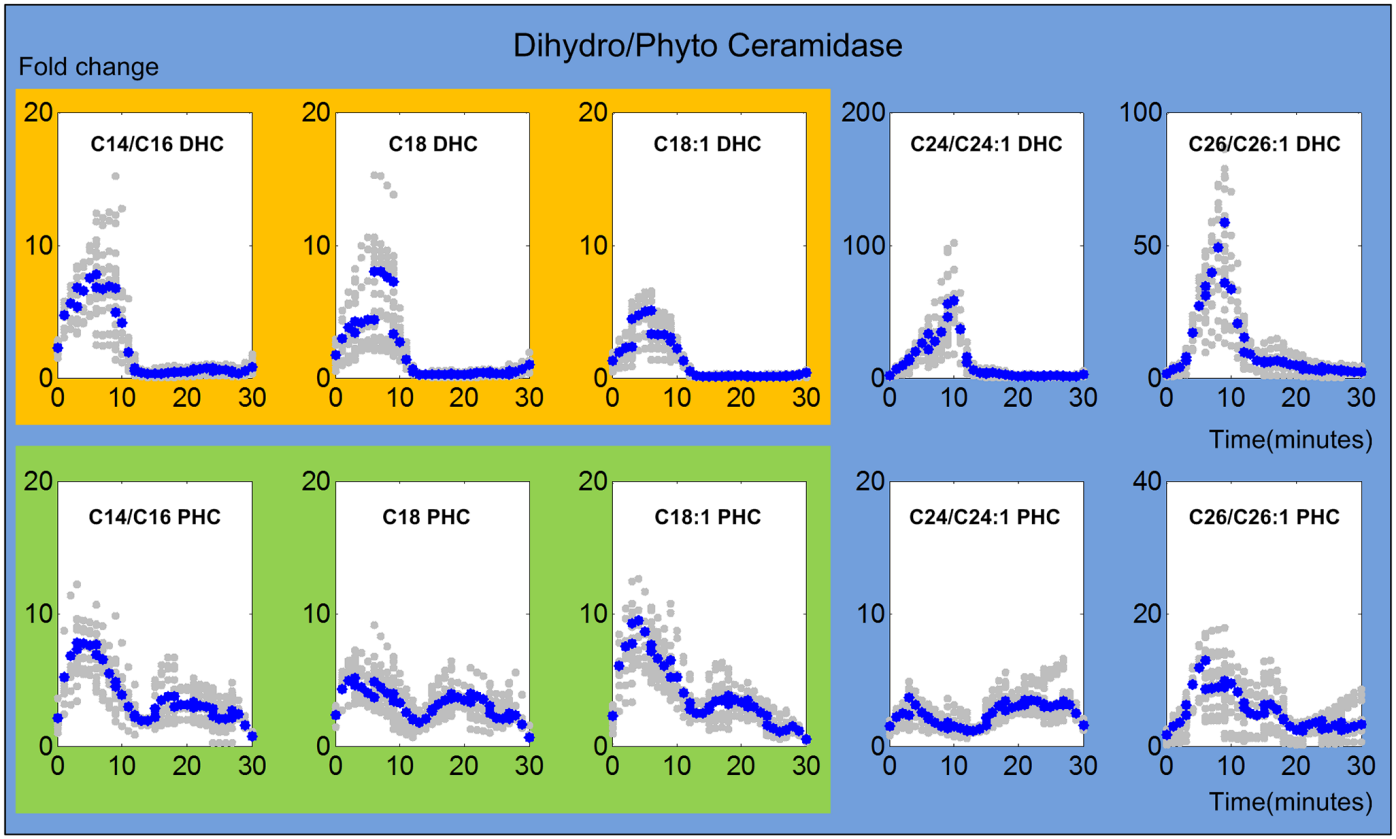
Trends in ceramidase activities. The upper and lower panels show activity trends of dihydroceramidase and phytoceramidase, respectively. The X-axis represents the 30 minutes of the heat stress experiment, while the Y-axis represents the fold changes in activities. In each plot, the blue and gray dots represent averaged and individual enzyme activities, respectively. The yellow and green shading marks similar enzyme activity patterns involved in DHC (top panel) and PHC (bottom panel) utilization, respectively.

Ceramidase catalyzes the reaction from ceramide to its sphingosine backbone. In our model, dihydroceramidase and phyotoceramidase are involved in five reactions that convert dihydroceramide species to dihydrosphingosines and phytoceramide species to phytosphingosines, respectively. Like the ceramide synthases, dihydroceramidase and phytoceramidase are activated immediately upon heat stress, as it is shown in Fig 5. This result indicates the important fact that heat stress not only triggers the production of sphingolipids but also their utilization, and that these two events occur almost simultaneously.

The enzyme activities of ceramidases can be grouped according to the DHC or PHC substrates they use. For dihydroceramidase, reactions involving C14/C16, C18, and C18:1 DHC as substrates show similar enzymatic activity patterns, which immediately rise, but stop being active after 12 minutes (shaded yellow in Fig 5). The reactions involving C24/C24:1 and C26/C26:1 DHC initially seem to change more slowly and peak slightly later than the enzymes for shorter-length substrates. For phytoceramidase, similar trends are observed for all chain lengths except C24 (shaded green in Fig 5), with peaks at about 5 minutes and thus a little later than for DHC. The pattern with regard to C24 PHC is not very distinctive, but seems to peak late into the heat stress. Note again that the absolute magnitudes of fold changes are to be considered with caution.

#### 3. IPC synthase

**Fig 6:**
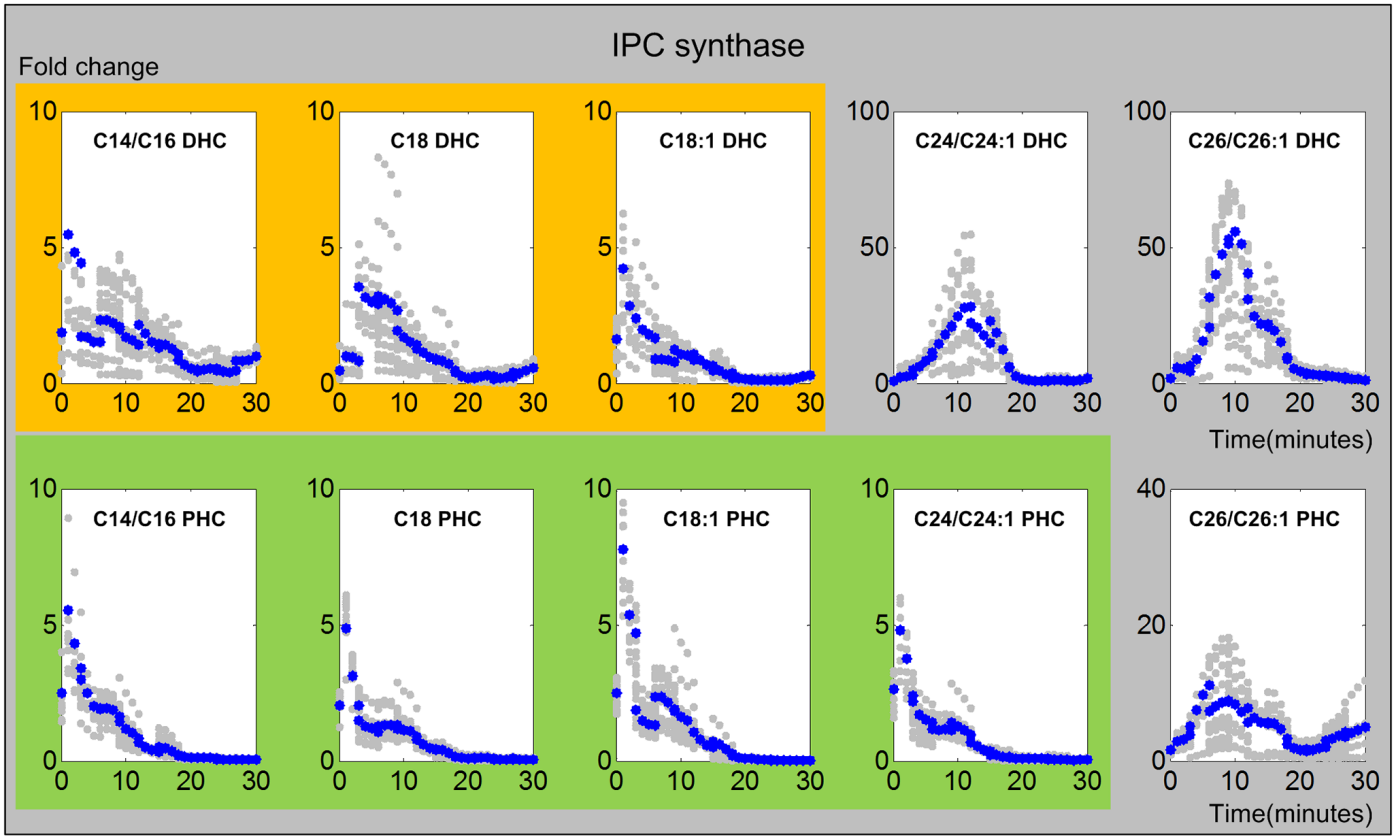
Trends in IPC synthase activities. The upper and lower panels show IPC synthase activities from DHC to IPC and from PHC to IPC, respectively. The X-axis represents the 30 minutes of the heat stress experiment, and the Y-axis represents the fold change in activities. In each plot, the blue and gray dots represent averaged and individual enzyme activities, respectively. The yellow and green shading marks similar enzyme activity patterns involved in DHC (top panel) and PHC (bottom panel), respectively.

IPC synthase controls a different pathway of ceramide utilization, which results in the synthesis of complex sphingolipids that are members of the IPC family. For long chain (C14 –C18) DHC, an initial activation of IPC synthases is again followed by low activity profiles during the last 10 to 15 minutes (Fig 6). The same is observed for C14 –C24 PHC. By contrast, activities associated with very long C24 –C26 DHC and C26 PHC peak between 10 and 15 minutes. The activities for DHC return to low values within the experimental time period, while the activity with respect to C26 PHC is still increased after 30 minutes.

#### 4. ISC1/IPCase

**Fig 7:**
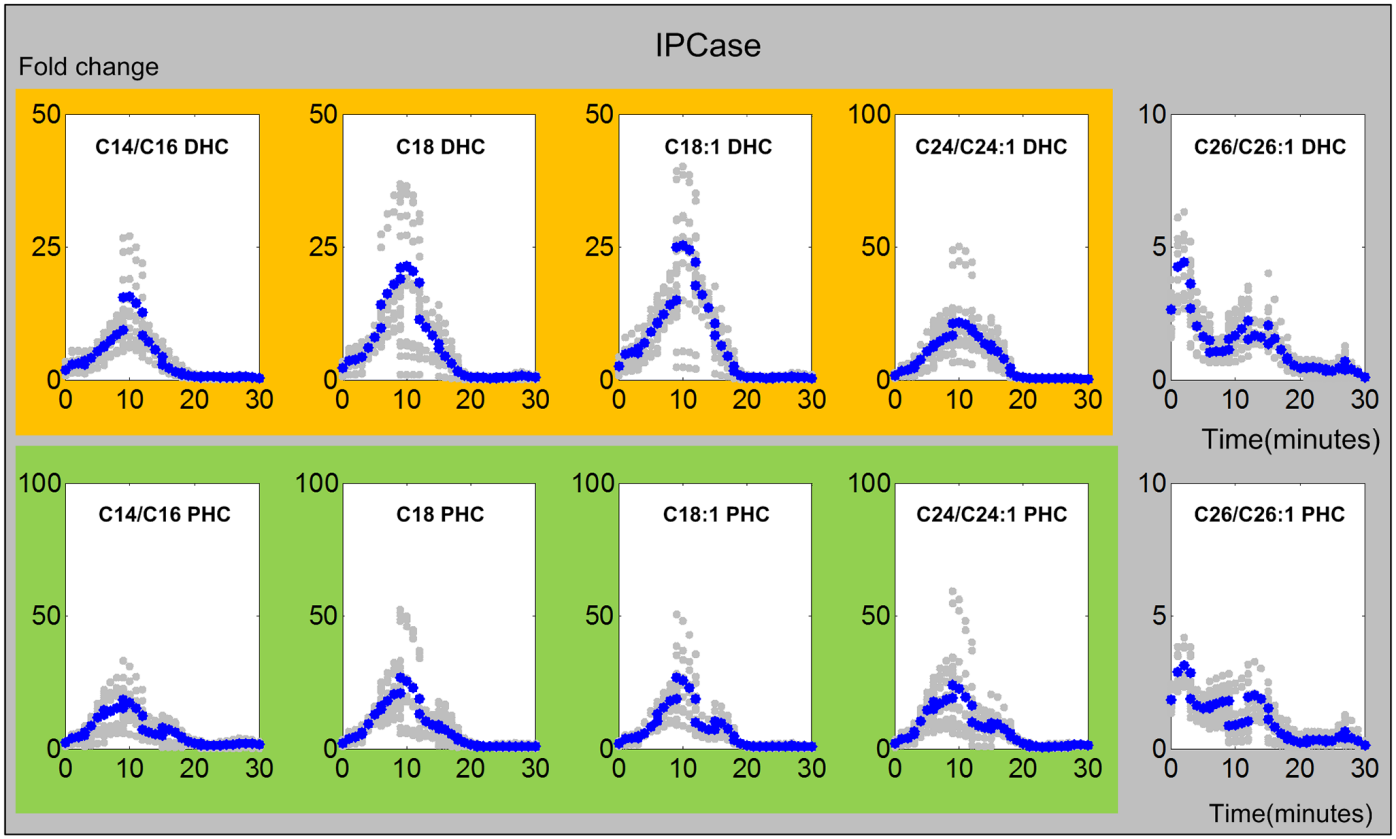
Trends in IPCase activities. The upper and lower panels show IPCase activities from IPC to DHC and from IPC to PHC respectively. The X-axis represents the 30 minutes of the heat stress experiment, and the Y-axis represents the fold change in activities. In each plot, the blue and gray dots represent averaged and individual enzyme activities, respectively. The yellow and green shading marks similar enzyme activity patterns involved in DHC (top panel) and PHC (bottom panel) side, respectively.

ISC1p breaks IPC into ceramides and thus constitutes another important process affecting the production of DHC and PHC in yeast. Fig 7 presents the inferred Isc1 activities. In all cases except for C26, the enzyme activities gradually increase upon heat stress and peak at about 10 minutes, before they return to the baseline. By contrast, the production of the very long chain (C26/C26:1) DHC and PHC are very low throughout the experiment.

#### 5. Dihydroceramide hydroxylase

**Fig 8:**
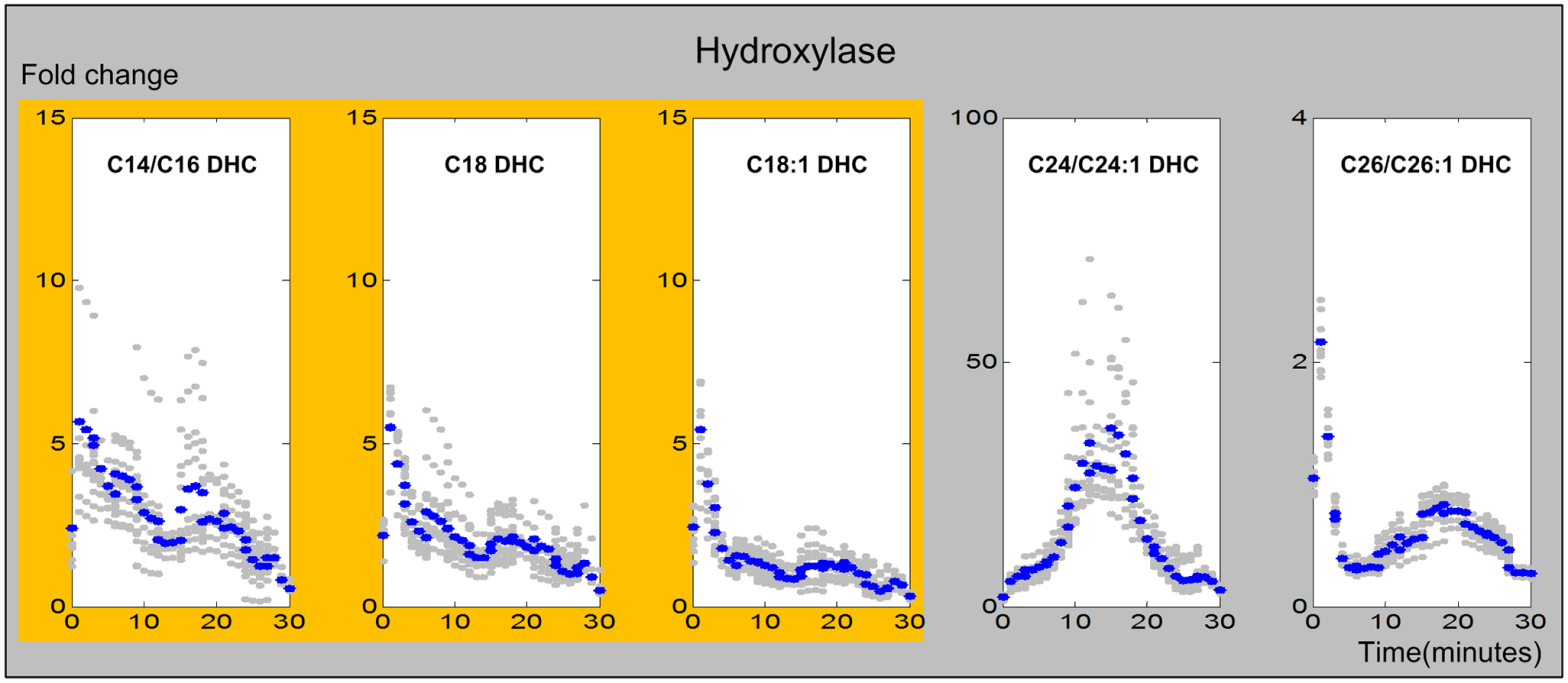
Trends in DHC hydroxylase activities. The X-axis represents the 30 minutes of the heat stress experiment and Y-axis represents fold-changes in activities. In each plot, the blue and gray dots represent averaged and individual enzyme activities, respectively. The yellow shading marks similar DHC hydroxylase activities from DHC toward PHC.

DHC hydroxylase catalyzes the reaction from DHC to PHC. In our model, five reactions, associated with different substrate chain lengths, are catalyzed by this enzyme system. The inferred enzymatic activities are shown in Fig 8. Except for the initial burst that was also observed for other enzymes, reactions with long chain fatty acids show similar and rather low enzymatic activities. By contrast, reactions involving very long chain fatty acid based DHC exhibit rather different dynamics. For C24/C24:1 DHC, the DHC hydroxylase is activated much later, and more strongly, than most other reaction steps, while for the C26/C26:1 substrate, the activity exhibits an immediate peak, as well as a second, low peak after about 15 to 20 minutes.

#### 6. Other enzymes

**Fig 9:**
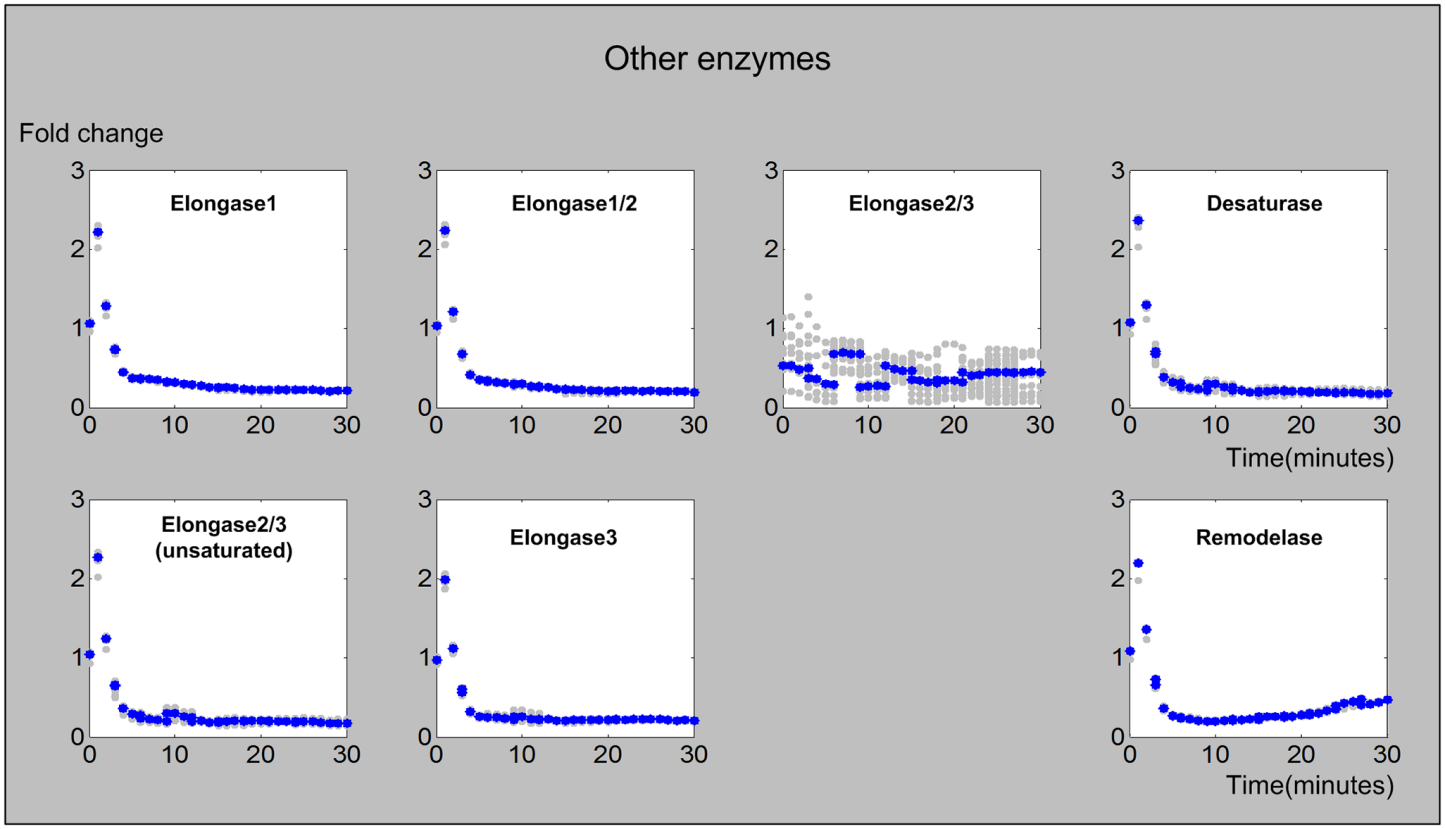
Activities of fatty acid elongases, desaturase, and remodelase. The X-axis represents the 30 minutes of the heat stress experiment and the Y-axis represents fold-changes in activities. In each plot, the blue and gray dots represent averaged and individual enzyme activities, respectively.

Fatty acid elongation is crucial for the backbone of ceramide biosynthesis, because it supplies the necessary fatty acyl CoAs of different lengths, from C14 to C26. As no time series measurements are available for the fatty acyl CoAs under heat stress, we assumed that these fatty acyl CoAs only show limited activation or degradation around their steady state. The corresponding enzyme activities in this elongation processes and also other accessory enzyme activities (remodelase, desaturase) are presented in Fig 9.

## Discussion

Recent research has indicated that yeast ceramides of different types and with different fatty-acyl chain lengths can exert distinct signaling functions [15]. It is therefore important to investigate how yeast cells manage to make the different ceramide variants available when needed. Our results shed light on this question. The computational inferences of dynamic enzymatic activities in ceramide biosynthesis are in agreement with previous insights into the control of sphingolipids under heat stress, but have a much higher resolution. The earlier results had suggested that essentially all enzymes in the pathway are involved in the adjustment of sphingolipid metabolism under heat stress, rather than just a few key enzymes, as one might have expected. The same observation holds again for the results obtained here, where we zero in on the much smaller but very important ceramide subsystem. Our simulations suggest that, for each ceramide species, the accumulation or utilization is governed by all associated enzymes. The results also render it evident that the ceramide heat stress response is not a haphazard panic reaction by the cell, but a well-coordinated, highly cooperative adaptation whose implementation is shared among all associated enzymes.

The experimental metabolic time series data and our customized optimization method allowed us to infer trends in all five major enzymes: ceramide synthase, ceramidase, IPC synthase, IPCase, and DHC hydroxylase. Interestingly, these enzyme systems exhibit distinct dynamic activity patterns that strongly depend on the carbon chain lengths of their fatty acids.

The initial activation of ceramide synthase confirms experimental and computational results indicating that heat stress induces the *de novo* biosynthesis of ceramides. In particular, our earlier results had shown that the production of dihydrosphingosine (DHS), the precursor of DHC, is immediately and strongly triggered by heat stress. The results here indicate that some of this newly synthesized DHS is directly channeled into long chain DHC. After just a few minutes, the activity of ceramide synthase for C14-C24 returns to the former baseline under optimal temperature conditions. Especially for C18:1 and C24, a second peak is detected after about 20 minutes, which coincides with the time when long chain DHC ceramidases cease to be active. The synthesis of the corresponding PHC increases more slowly and peaks about six to eight minutes into the heat stress. This short delay between DHC and PHC synthesis may be explained with the fact that phytosphingosine, the substrate of PHC synthase, must first be produced from DHS. In contrast to these patterns, the activities for very long chain substrates (C26 DHC and PHC) only flare up very briefly, but strongly, and then remain rather low. Although this activity trend is short-lived, a comparison with our earlier results suggests that this pattern actually dominates the overall trend in ceramide synthase. Indeed, this suggestion aligns well with the fact that C26 PHC is by far the most prevalent ceramide variant under normal conditions (Fig 3).

By catalyzing the utilization of complex sphingolipids, IPCase is the second source for ceramides. In contrast to *de novo* biosynthesis, these processes initially increase much more slowly and exhibit a strong and long lasting activity peak between about 5 and 20 minutes (Fig 7). Subsequently, their activities essentially cease. Interestingly, the trends are very similar for DHC and PHC and for chain lengths up to 24, which may suggest that the same enzyme could catalyze all reactions for both DHC and PHC of chain lengths up to 24. The dynamics for C26 DHC and PHC is distinctly different.

The DHC hydroxylase reaction facilitates an internal redistribution between DHCs and PHCs. It is activated instantly for long chain DHCs, with a subsequent decrease in activity, whereas C24 activity occurs mostly between 10 and 20 minutes (Fig 8). The activities with respect to C26/C26:1 DHC show a mixed pattern with a very small magnitude.

The utilization of ceramides follows two routes, namely toward the sphingosine backbone via ceramidase, and toward IPC via IPC synthase. The long chain DHC ceramidases arguably exhibit the most striking pattern (Fig 5). Their activities rise immediately with heat stress and are sustained for about ten minutes, after which the activities drop quickly and cease altogether. As these activities are much higher than for the corresponding ceramide synthases, it appears that the cells are preferably channeling long chain material to DHS. The corresponding activities for PHC are not as clear-cut. They also increase, but not as quickly or strongly, and return to their baseline over the entire 30-minute time period. This difference between DHC and PHC substrates may again be explained with the fact that phytosphingosine and PHC must first be synthesized from dihydrosphingosine and DHC, respectively. Intriguingly, the activities for very long DHC substrates and for C26 PHC increase more gradually and peak between 5 and 10 minutes. As C26 PHC is the most prevalent ceramide species, this pattern dominates the overall trend in ceramidase.

Finally, IPC synthase incorporates ceramides into complex sphingolipids. Here, the activities for long chain DHC and PHC substrates rise instantly, and the activities essentially cease after about 20 minutes (Fig 5). By contrast, the highest use for very long chain DHC and PHC occurs at about 10 minutes of heat stress. As in other cases, these trends are in line with the overall trends we observed in our previous analysis.

Taken together, it appears that the immediate response to heat stress is the *de novo* synthesis of long chain DHC, its conversion into the corresponding PHC, and the return of some material to DHS. Also, some long chain DHCs and PHCs are incorporated into complex sphingolipids. Between 5 and 10 minutes of heat stress, long and very long chain PHCs are generated. Around 10 minutes into heat stress, complex sphingolipids are used to generate DHC and PHC of all lengths. Also at this time, C24/C24:1 DHC is converted into PHC. Most activities are back to normal at the end of the 30-minute period, which is consistent with our earlier findings [13].

The molecular and cell-physiological reasons for the differences in activity patterns towards substrates of different lengths are unknown. The most straightforward hypotheses might be that the differences are due to:

1. the existence of specific enzymes or isozymes for different substrates;
2. different affinities of the same enzymes to substrates with different N-acyl chain lengths;
3. compartmentalization of substrates and/or enzymes, which would allow the same enzyme to be regulated differently in its action on distinct substrates.

In mammalian cells, at least five ceramide synthases were identified [31], and they perform different but overlapping functions with respect to different fatty acyl CoAs. In yeast, LIP1, LAC1 and LAG1 are known as genes coding for subunits of ceramide synthase, but otherwise not much is known about the reactions associated with different ceramide species. Our computational inferences indicate that reactions using substrates with different fatty acyl chain lengths are grouped, often into long chain and very long chain classes, which could suggest that ceramide associated enzymes are regulated in a “fatty acyl chain length specific” manner. However, there is so far no evidence that different species of the enzymes in question, which seems to suggest that explanation (3) might be more likely than (1) and (2). Further experimental research will be needed to support or refute this conclusion.

Beyond the particular application to ceramide metabolism, the computational modeling and inference methods in this work demonstrate how metabolite profiles, obtained as time series data, may be used to decipher *in vivo* strategies with which cells organize their responses to environmental stimuli.

## Materials and Methods

### Experimental Data

Ceramide time series data for the present study were obtained *de novo*, as described in [32]. *S*. *cerevisiae* cells were cultured overnight at an optimal temperature of 30°. In duplicate experiments, the cells were moved to a water bath that was kept 39°C, which causes heat stress in yeast, and sampled every 5 minutes between 0 and 30 minutes. The samples were analyzed with High Performance Liquid Chromatography and Mass Spectrometry (HPLC-MS) to yield heat stress time series data of the following dihydro- and phyto-ceramide species: C14, C16, C18, C18:1, C20, C22, C24, C26; here the numbers refer to fatty acyl chain lengths and “:1” refers to an unsaturated fatty acid with one double bond. Considering that some datasets were missing or measurements fell below the detection limit, we used C14/C16, C18, C18:1, C24/C24:1 and C26/C26:1 DHC/PHC to construct our mathematical model.

**Fig 10:**
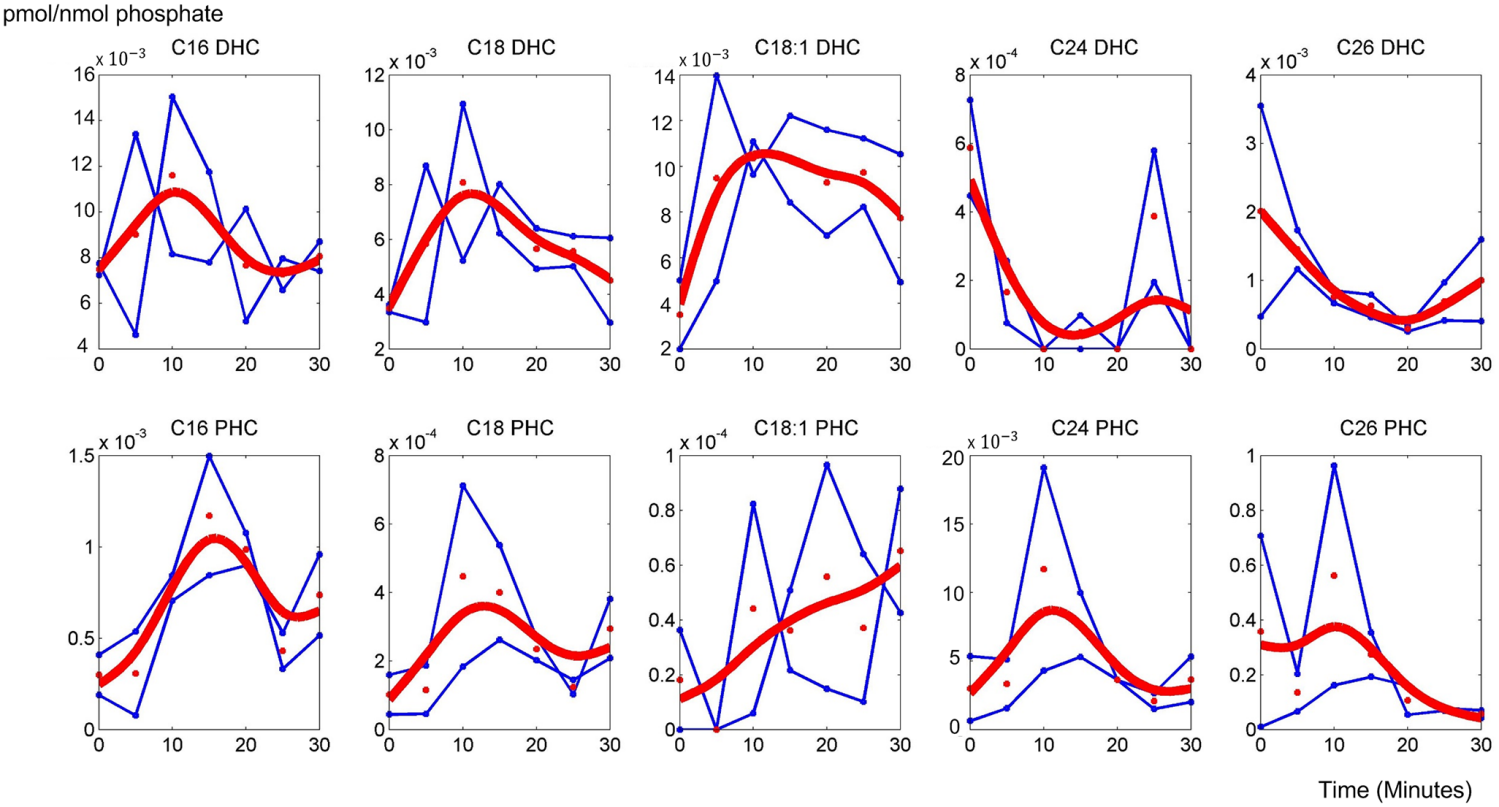
Raw duplicate time series concentrations of ceramide species (connected blue symbols) and their means (red symbols). The red curves show the smoothing spline interpolations of each dataset.

As in our previous study [13], we found it beneficial to interpolate the data in a smooth fashion. It seems reasonable to assume that the heat stress response is a continuous phenomenon, and that a minimally biased spline technique would reflect the true dynamics in acceptable approximation. The raw and smoothed, interpolated data are exhibited in Fig 10. One should note that the Y-axes in Fig 10 are quite different, which indicates a large variation in prevalence of the various ceramide species.

### Previous Work on Enzyme Activity Inferences

Yeast responds to heat stress within minutes. Among the different aspects to this response, the concentration profile of sphingolipids starts to change in less than two minutes. Due to the complexity of the pathway, these changes in synthesis and degradation, which result from activity changes in enzymes, can hardly be inferred with intuition alone. To shed light on the response strategy, we recently proposed a customized computational approach to infer the dynamic changes in enzymatic activities from sphingolipid time series data [13]. These data consisted of time course measurements that were taken under heat stress conditions every 5 minutes until the end of a 30-minute interval and contained concentration measurements of dihydrosphingosine (DHS), dihydrosphingosine 1-phosphate (DHS1P), phytosphingosine (PHS), phytosphingosine 1-phosphate (PHS1P), dihydroceramide (DHC) and phytoceramide (PHC).

In order to infer the heat-induced changes in enzymatic activities, we used a Generalized Mass Action (GMA) model, in which every process was represented as a product of a rate constant and of all variables that directly affected the process, raised to a power [24]. The specific model was adopted from our earlier work [22,23] and included 31 metabolites as dependent variables and 64 enzymes or cofactors as independent variables. With this model as base structure, we developed a piecewise optimization approach.

First, the time series measurements were interpolated by smoothing splines and then re-sampled to produce time series values for every minute during the experimental time period. These values were entered into the GMA model. For each time interval, we formulated and solved the optimization problem of finding a set of enzymatic activities to establish the lipid profiles in that specific time frame. Given 31 time points, we thus found 30 corresponding sets of enzymatic activities that generated the observed dynamic metabolic profiles. An additional randomization scheme allowed us to infer solution spaces and confidence bands rather than point estimates. Several validation studies confirmed the results. The trajectories of the computed enzymatic activities revealed interesting regulatory mechanisms of sphingolipid metabolism, as described in the introduction.

### Methodological Alterations for Explaining the Dynamics of Ceramide Variants

The basic concepts of the modeling method were taken from our previous study [13]. As before, we smoothed the heat stress time series data of the six DHC and PHC species with different chain lengths, considered one-minute time intervals, and developed a customized, piecewise optimization approach that allowed us to infer changes in enzyme activities in a step-by-step manner. Also as in earlier studies, the system was represented as a GMA model. The pathway system under investigation is depicted in Fig 2. It consists of three major parts: synthesis and utilization of DHC, synthesis and utilization of PHC, and fatty acid elongation.

In comparison to the previous inference of all sphingolipid enzyme activities, the subsystem we consider here is relatively small. However, by accounting for the different chain-length variants, the system is much more detailed and leads to surprising complexity. In particular, the fatty acid elongation process becomes critical, whereas it was modeled only coarsely in the earlier analysis. It is our goal here to tease out the details of fatty acid elongation and the synthesis and degradation of variant ceramide species with different fatty acyl groups.

All responses outside the subsystem addressed here are expected to be the same as in the larger system, within normal biological variability, which allows us to use the previous model as a large set of dynamic boundary constraints that govern the sphingolipid system at large. For instance, fluxes entering the ceramide subsystem are directly taken from the large sphingolipid model. Similarly, the heat stress concentration of palmitoyl fatty acyl CoA can be directly imported from the sphingolipid model. The table in Fig 2 summarizes constraints on fluxes in the present model, imposed by the prior model.

The resulting ceramide subsystem has 53 fluxes and 15 dependent variables (Fig 2). *X*_1_ to *X*_5_, *X*_6_ to *X*_10_, and *X*_11_ to *X*_15_ represent the corresponding species of C16-DHC to C26-DHC, C16-PHC to C26-PHC, and C16-fatty acyl CoA to C26-fatty acyl CoA, respectively.

**Fig 11:**
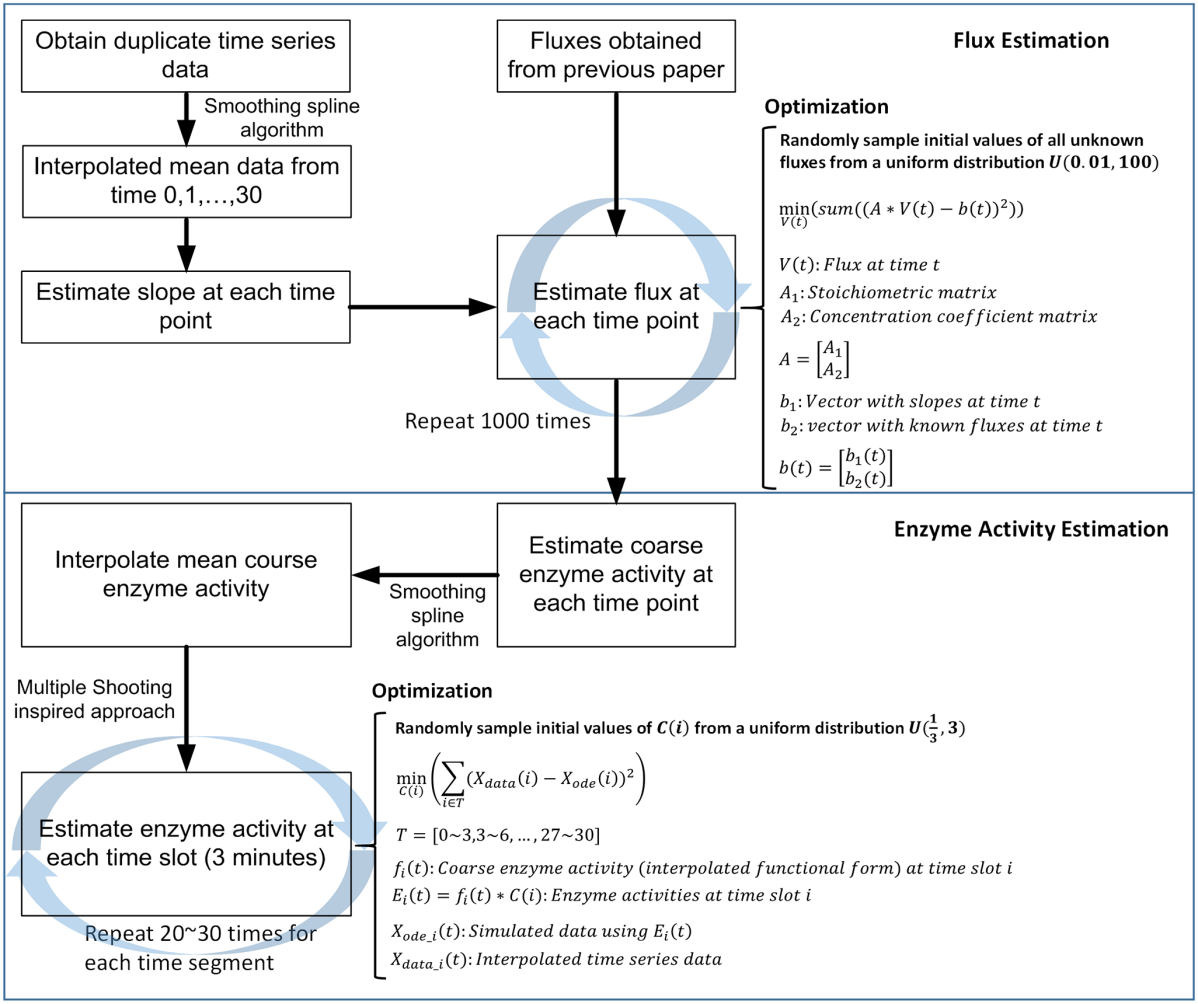
Details of the procedures of flux estimation and enzyme activity estimation.

Details of the two core components of our approach, namely the estimation of dynamic fluxes and of enzyme activities, are shown in the flowchart of Fig 11. The first task, as shown in the upper panel of Fig 11, consists of checking the mass balances within the system and to construct the stoichiometric matrix that describes the production and degradation rates of the dependent metabolites. Because the system has considerably more fluxes than metabolites, we are faced with a highly underdetermined system. We are dealing with this situation by solving the system in 30 pieces, starting from the initial steady state to time 1, from time 1 to time 2, all the way to the end of the heat stress experiment (30^th^ minute). The following describes in more detail a customized optimization strategy with which we determine the flux distribution in each time point.

Each flux is determined such that the model as a whole matches the observed data within a sufficiently small range of noise. Furthermore, all subsets of these fluxes, for instance, those representing the DHC hydroxylases, must collectively be consistent with the known fluxes of our previous model (see table in Fig 2). Also, all fluxes should change relatively smoothly from one time interval to the next. Finally, all fluxes are subject to upper and lower bounds. These tasks are formulated as a constrained nonlinear optimization program. Specifically, at each time point, we use two types of constraints. First, we constrain the system by ensuring that the slopes of the dependent variables (*X*) at a given time point *t* are sufficiently close to *X*(*t*) − *X*(*t* − 1), which we accomplish by minimizing the sum of squared errors between these differences and the corresponding slopes of these *X* variables. Second, we constrain the system by ensuring that the fluxes entering the system from the outside are collectively equivalent to those of the former model (*cf*. Table in Fig 2). These two constraints can be formulated as a combined, single objective function. To achieve robustness of the solution, the system is solved repeatedly by assigning for all unknown fluxes initial values that are drawn randomly from the uniform distribution U(0.01, 100). The results suggested that 1,000 simulations return sufficiently diverse ensembles.

One could surmise that the rather strong variability in the time series data (Fig 10) would unduly affect the flux estimation. To test this hypothesis, we estimated fluxes based on interpolated concentration data that were perturbed in either direction by a random factor sampled from U(1/1.5, 1.5). We compared these estimated fluxes with those obtained from noise free interpolated data. The flux distributions show very similar patterns (Fig S4.1 and S4.2 in S5 Text); detailed procedures are provided in the *Supplements*.

**Fig 12:**
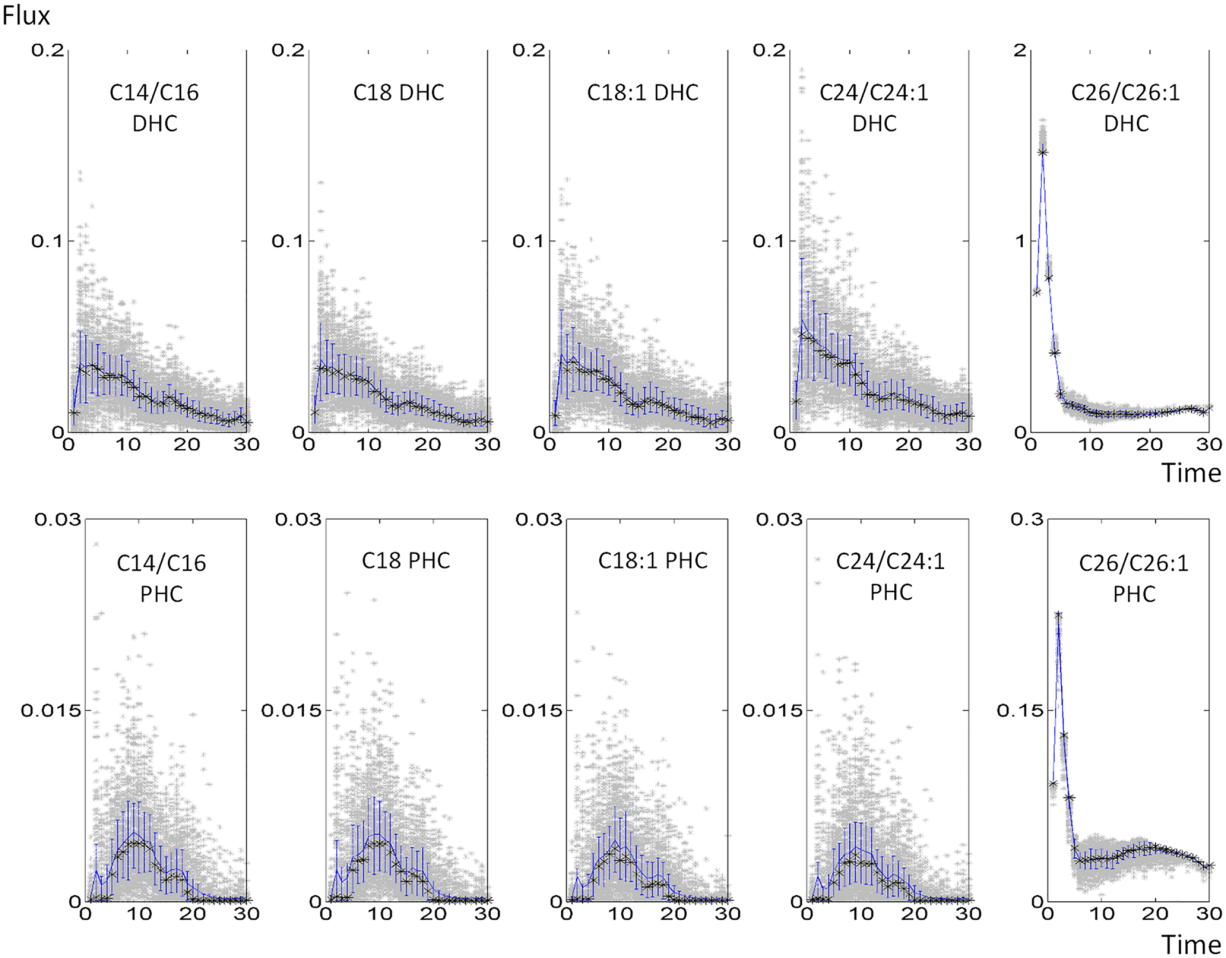
Dynamic flux distributions of 10 reactions catalyzed by ceramide synthase. Each grey dot represents one simulation. The blue line indicates the means, the black asterisks show the medians, and blue bars represent 20^th^ and 80^th^ percentiles (blue bars).

**Fig 13:**
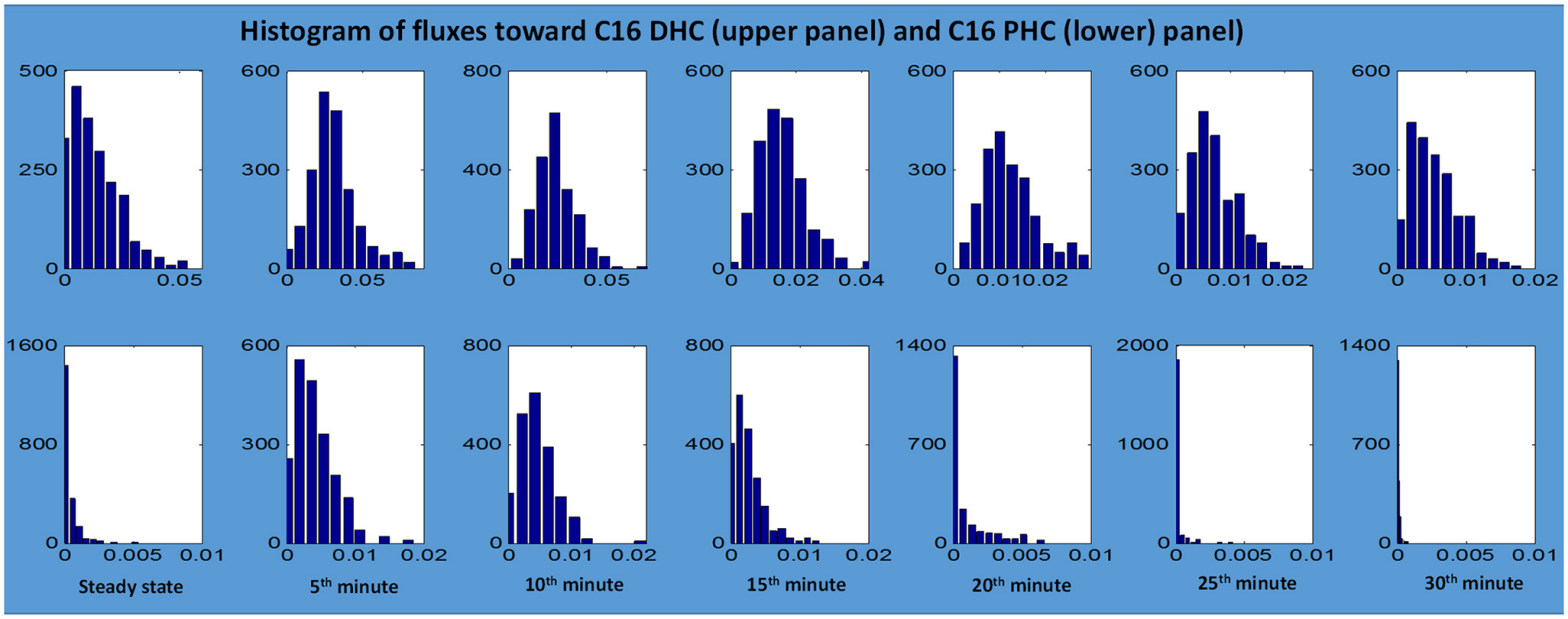
Histograms of ceramide synthase fluxes from C16 DHS to C16 DHC (upper panel) and from C16 PHS to C16 PHC (lower panel). Time 0 represents normal steady-state temperature conditions at the beginning of the heat stress experiment, which lasts for 30 minutes.

As an example, the resulting fluxes of reactions catalyzed by ceramide synthase are given in Fig 12; other fluxes are shown in the *Supplements* (Fig S1 in S2 Text). Once the flux distributions are computed for each time point, we examine the histogram of each flux in each time point to ensure that the solutions are well constrained; details are presented in the *Supplements* (Fig S3 in S4 Text). The analysis revealed that most of the flux distributions at any given time point were rather tightly bell shaped, suggesting the use of the mean value of each flux at each time point as an appropriate, time-dependent estimate (Fig 13). To validate this result further, we compared the sum of squared errors (SSEs) between the 1,000 individual fluxes and the corresponding averaged fluxes at each time point. The SSE of the averaged flux always falls within the range of SSEs from individual fluxes (Fig S5 in S6 Text); further details are provided in the *Supplements*. We also redid the analysis with medians, but the results were essentially the same.

**Fig 14:**
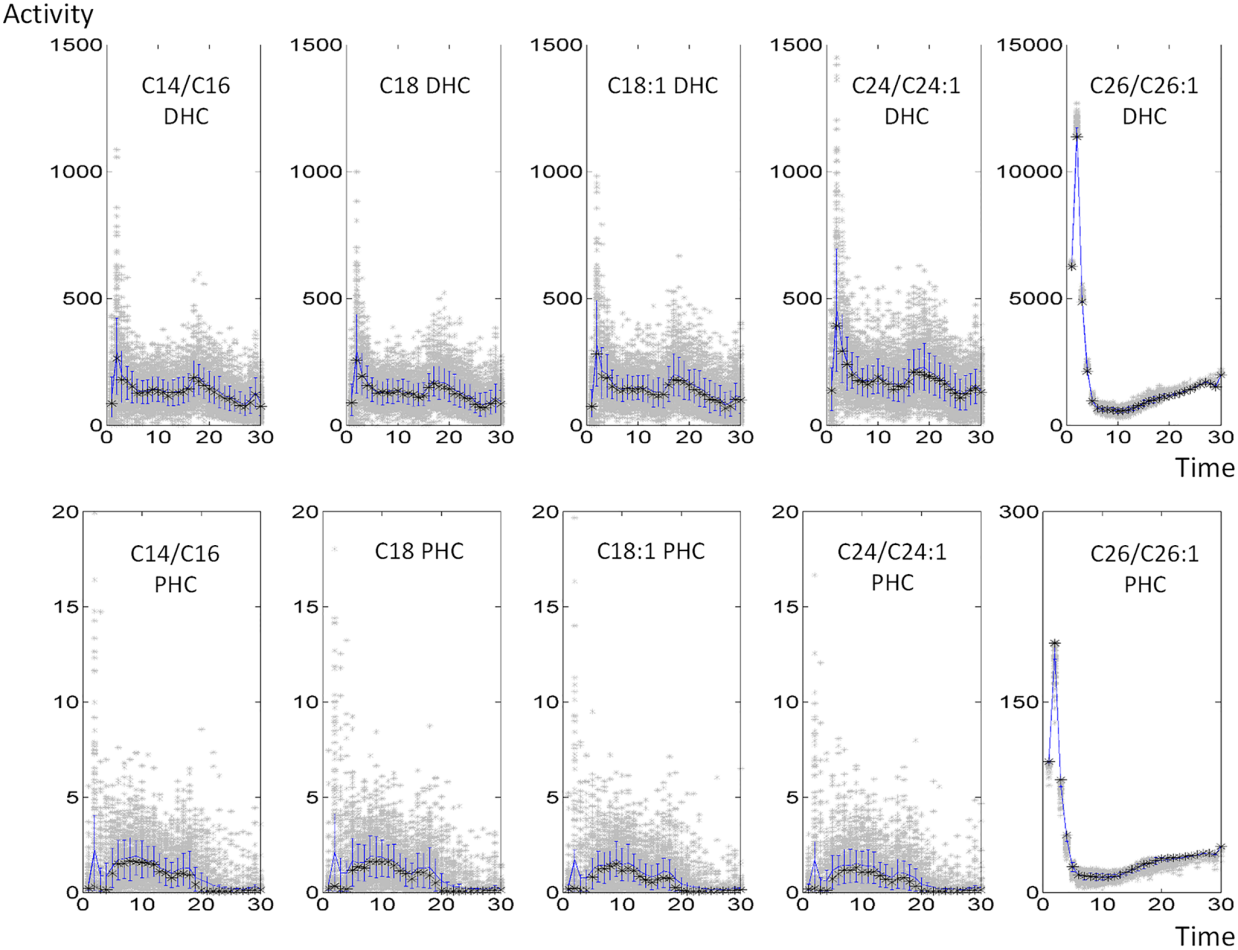
Rough estimation of ceramide synthase activities. Each grey dot represents one simulation. The blue line indicates the means, the black asterisks show the medians, and blue bars represent 20^th^ and 80^th^ percentiles (blue bars).

The method of Dynamic Flux Estimation [33] allows us to obtain important hints for how material flows within the system. The resulting flux estimates are also important for constructing a dynamic model of ceramide synthesis and utilization upon heat stress. For later purposes, we need to identify how substrates, enzyme, modulators and kinetic parameters contribute to the magnitude of a flux. Because the actual enzyme amounts and rate constants are not known, we assume, as it is commonly done, that enzyme activities enter a flux representation in a linear manner and that substrates contribute with a power of 1, which corresponds to a mass-action formulation. Given these assumptions we obtain coarse estimates of the product of the rate constant and the enzyme activity. This product is similar to a *V*_*max*_ value, which by definition consists of the product of *k*_*cat*_ and the total enzyme concentration. Some of these estimates are shown in Fig 14 as functions of time. (All estimates are presented in Fig S3 in S4 Text)

Based on these time dependent estimates of flux magnitudes, we employed smoothing splines with proper degrees to obtain smooth time trends in enzyme activities (please refer to lower panel in Fig 11). The functional representations of enzyme activities were entered into the ODE model in order to obtain trends in the different ceramide species. Under ideal conditions, these trends should match the observed concentration profiles. By using the residual errors between the results of the described modeling strategy and the data, we created an optimization strategy that iteratively refined the trends in enzyme activities. This strategy was gleaned from the method of Multiple Shooting, which is a well-documented methodology for fitting dynamical data in boundary value problems [34,35]. While most optimization methods that correspond to a single shooting strategy aim at searching for one parameter set to fit the observed dynamic trajectory in its entirety, multiple shooting splits the time series into successive time frames and initially searches for independent parameter sets that match the data one frame at a time. In many cases of complex dynamic systems, this type of multiple shooting has demonstrated a better performance than single shooting. Here, we address a separate initial value problem for each time frame, and the last data point in each time frame is not defined as a condition for the subsequent frame.

We applied the multiple-shooting inspired strategy to fit the various ceramide time series data. However, instead of searching for entirely new parameter sets for each time frame, the task here is simpler, because the algorithm searches merely for slight adjustments of the coarse functional forms of enzyme activities that we had previously derived for each time frame to fit the ceramide data. Specifically, we associated unknown coefficients *C* to the enzyme activities in the ODEs. For example, from the 3^rd^ to the 6^th^ minute of heat stress, the functional representation of the enzyme activity, *f*_3−6minute_ (*t*), was replaced with *C*_3−6minute_ * *f*_3−6minute_ (*t*). With all coefficients set equal to one, the ODEs are unchanged. However, by using the coefficients as free parameters, we are now capable of adjusting the system dynamics in each time frame. Thus, we subdivided the 30-minute time frame of the heat stress experiment into 3-minute intervals and fit the data separately in each interval. Furthermore, to minimize bias, we executed the search algorithm with many random initial settings for each coefficient, each enzyme, and each timeframe so that we obtained ensembles of solutions within a larger solution space. The slightly adjusted enzyme activities were then collected for biological inference.

## Supporting Information

S1 Text Model equations. (DOCX)

S2 Text Flux distributions. (DOCX)

S3 Text Magnitudes of enzyme activities. (DOCX)

S4 Text Histograms. (DOCX)

S5 Text Impact of data variability on estimated distributions of dynamic fluxes. (DOCX)

S6 Text Justification for using averaged fluxes in the inference of enzyme activities. (DOCX)
