# Supplementary material for: Dynamics of the Heat Stress Response of Ceramides with Different Fatty-Acyl Chain Lengths in Baker’s Yeast
Source: PLoS Comput Biol. 2015 Aug 4;11(8):e1004373. doi: 10.1371/journal.pcbi.1004373 (PMC4524633; doi:10.1371/journal.pcbi.1004373)
Supplement: S1 Text — (DOCX) [file pcbi.1004373.s001.docx]

**Supplements**

**Dynamics of the Heat Stress Response of Ceramides with Different Fatty-Acyl Chain Lengths in Baker’s Yeast**

**Po-Wei Chen, Luis L. Fonseca, Yusuf A. Hannun, Eberhard O. Voit**

**S1 Text: Model equations**

The model equations below correspond to the pathway in Fig. 2 of the main article.$X_{1}\sim X_{5}$ represent C14/C16, C18, C18:1, C24 and C26 DHC, respectively, while $X_{6}\sim X_{10}$ represent C14/C16, C18, C18:1, C24 and C26 PHC, respectively. $X_{11}\sim X_{15}$ represent the corresponding C14/C16, C18, C18:1, C24 and C26 fatty acyl CoA variants. DHS and PHS were coded as $X_{16}$ and $X_{17}$. Each $V_{i}$ denotes a flux, which is modeled according to the Generalized Mass Action (GMA) framework within Biochemical Systems Theory[[1](#_ENREF_1)]. For example, $V1=\gamma_{1,1}X_{16}^{f_{1,1,16}}X_{11}^{f_{1,1,11}}$. In this formulation, $\gamma_{1,1}$ is a rate constant, and $f_{1,1,16}$ and $f_{1,1,11}$ are kinetic orders. The rate constants are at first unknown and must be specified from the data, while the kinetic orders were obtained from the previously published paper or assumed to have a value of 1, if they were unknown. $X_{16}$ and $X_{17}$ (DHS and PHS) are independent functions obtained from polynomial fitting of the data from published paper [[2](#_ENREF_2)].

$$\frac{dX_{1}}{dt}=V1+V2-V3-V4-V5$$

$$\frac{dX_{2}}{dt}=V6+V7-V8-V9-V10$$

$$\frac{dX_{3}}{dt}=V11+V12-V13-V14-V15$$

$$\frac{dX_{4}}{dt}=V16+V17-V18-V19-V20$$

$$\frac{dX_{5}}{dt}=V21+V22-V23-V24-V25$$

$$\frac{dX_{6}}{dt}=V4+V26+V27-V28-V29$$

$$\frac{dX_{7}}{dt}=V9+V30+V31-V32-V33$$

$$\frac{dX_{8}}{dt}=V14+V34+V35-V36-V37$$

$$\frac{dX_{9}}{dt}=V19+V38+V39-V40-V41$$

$$\frac{dX_{10}}{dt}=V24+V42+V43-V44-V45-V46$$

$$\frac{dX_{11}}{dt}=V47-V1-V26-V48$$

$$\frac{dX_{12}}{dt}=V48-V6-V30-V49-V50$$

$$\frac{dX_{13}}{dt}=V49-V11-V34-V51$$

$$\frac{dX_{14}}{dt}=V50+V51-V16-V38-V52$$

$$\frac{dX_{15}}{dt}=V52-V21-V42-V53$$

**References**

1. Savageau, M.A., *Biochemical systems analysis. I. Some mathematical properties of the rate law for the component enzymatic reactions.* Journal of Theoretical Biology, 1969. **25**(3): p. 365-369.

2. Cowart, L.A., et al., *Revealing a signaling role of phytosphingosine-1-phosphate in yeast.* Mol. Syst. Biol., 2010. **6**: p. 349.
