# Supplementary material for: Dynamics of the Heat Stress Response of Ceramides with Different Fatty-Acyl Chain Lengths in Baker’s Yeast
Source: PLoS Comput Biol. 2015 Aug 4;11(8):e1004373. doi: 10.1371/journal.pcbi.1004373 (PMC4524633; doi:10.1371/journal.pcbi.1004373)
Supplement: S2 Text — (DOCX) [file pcbi.1004373.s002.docx]

**Supplements**

**Dynamics of the Heat Stress Response of Ceramides with Different Fatty-Acyl Chain Lengths in Baker’s Yeast**

**Po-Wei Chen, Luis L. Fonseca, Yusuf A. Hannun, Eberhard O. Voit**

**S2 Text: Flux distributions**

The distributions of fluxes contributing to the dynamics of the key sphingolipid species addressed in this paper are given in the main text. Figure S1 provides the remaining flux distributions. Each set of results was collected from 2,000 simulations. Gray dots, blue lines, blue bars and black asterisks represent single simulation results, mean values, 20^th^ and 80^th^ percentiles and median values, respectively.

**Ceramide synthase**


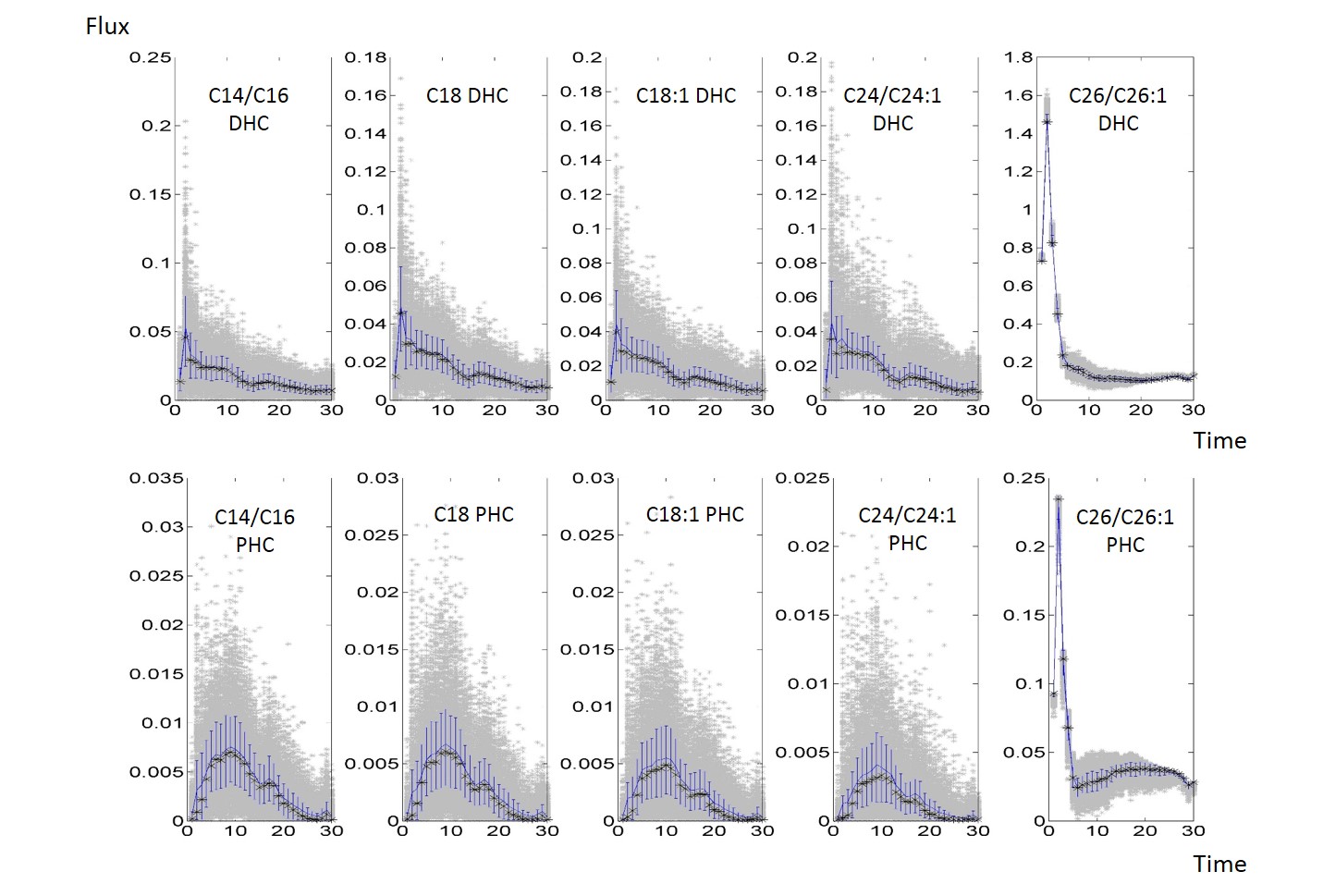


**Dihydroceramidase & Phytoceramidase**

**
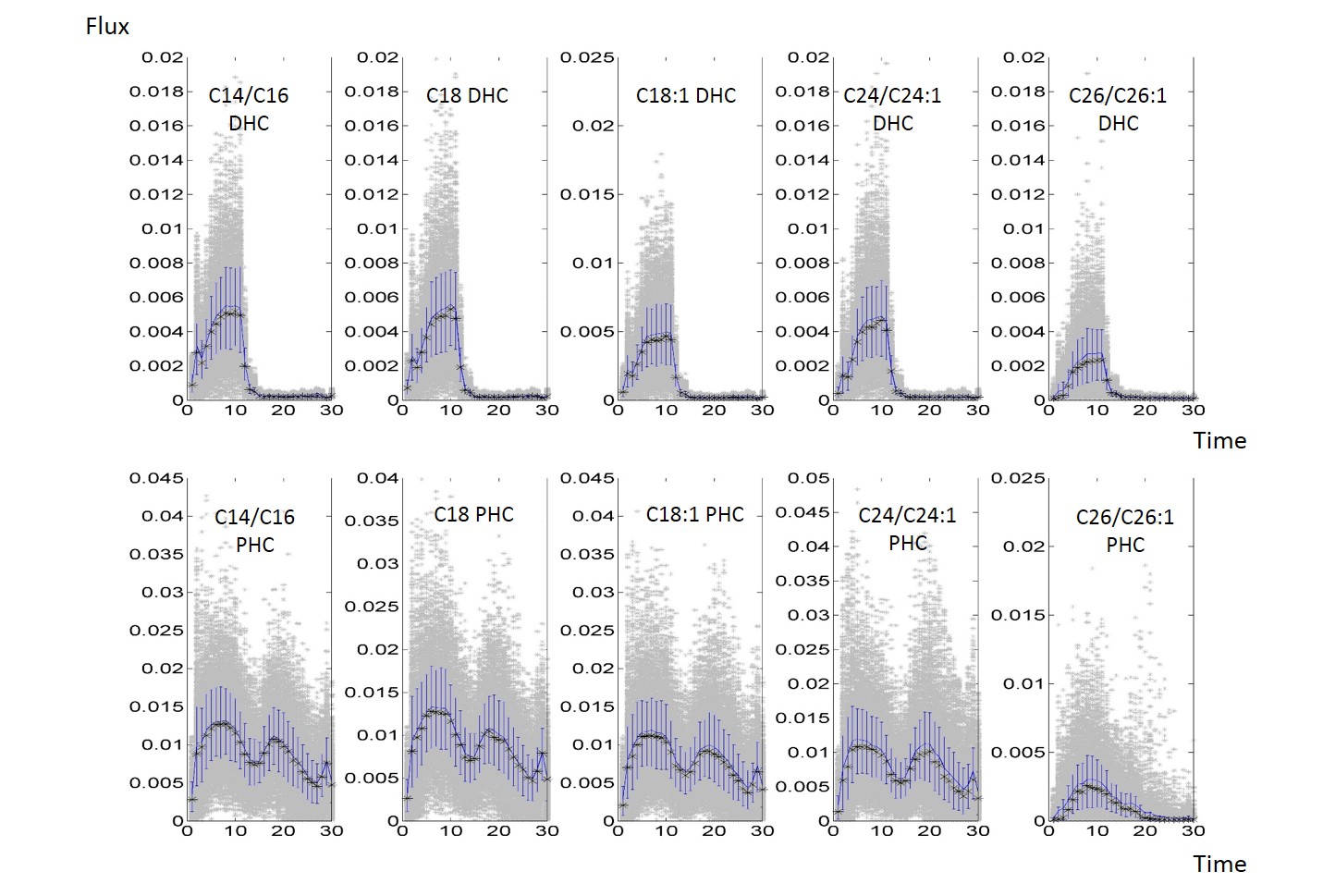
**

**IPC synthase**

**
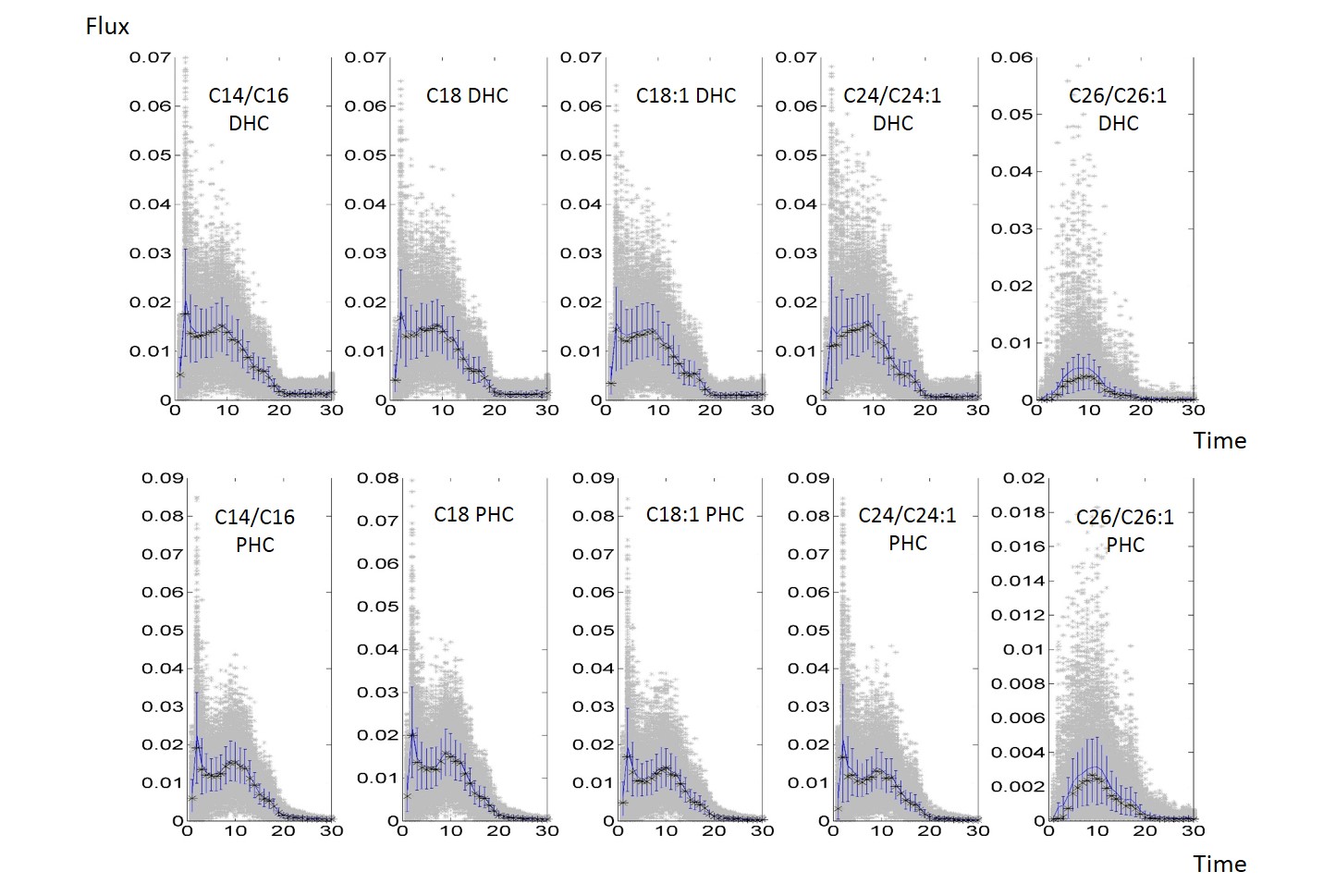
**

**IPCase**

**
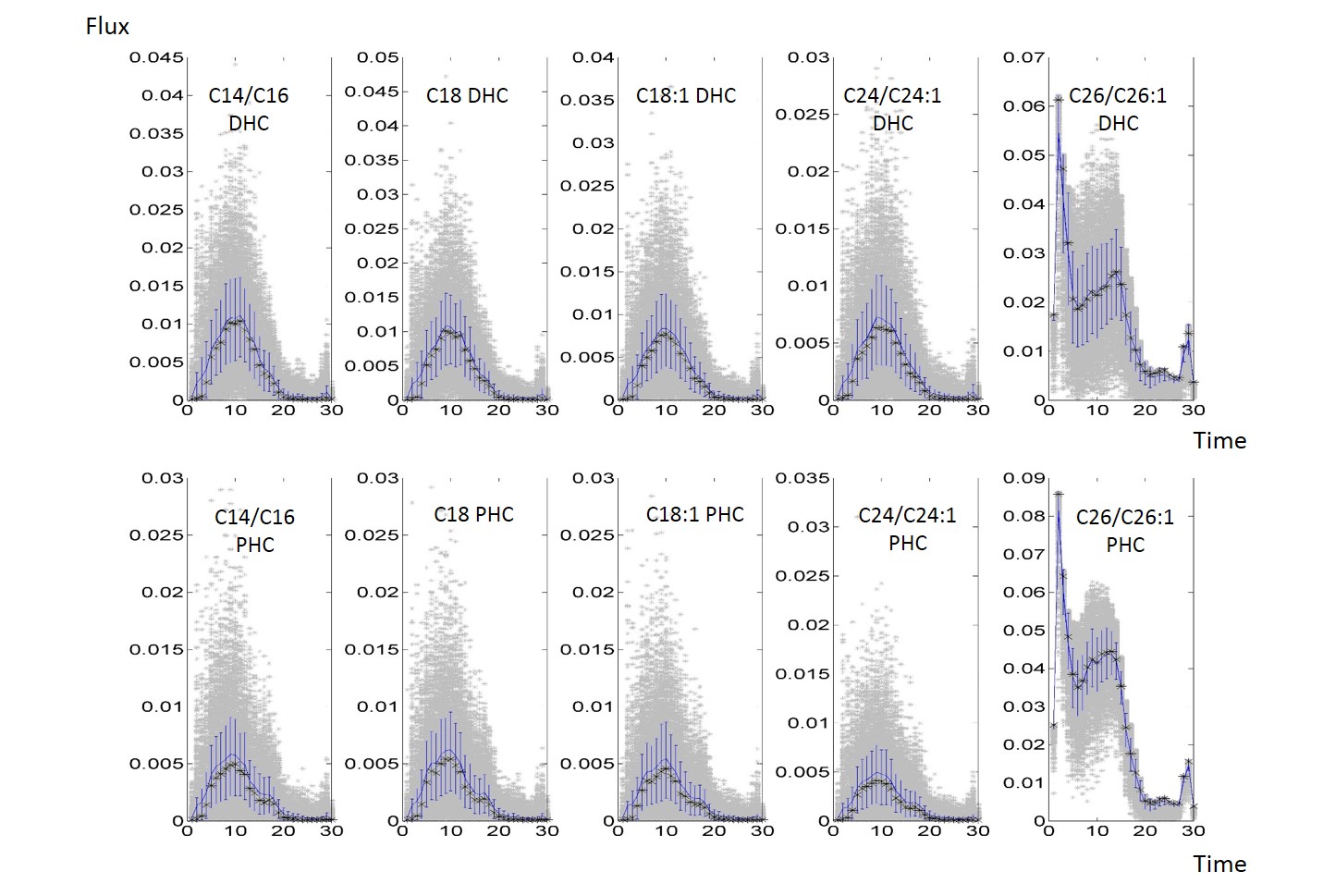
**

**Hydroxylase**

**
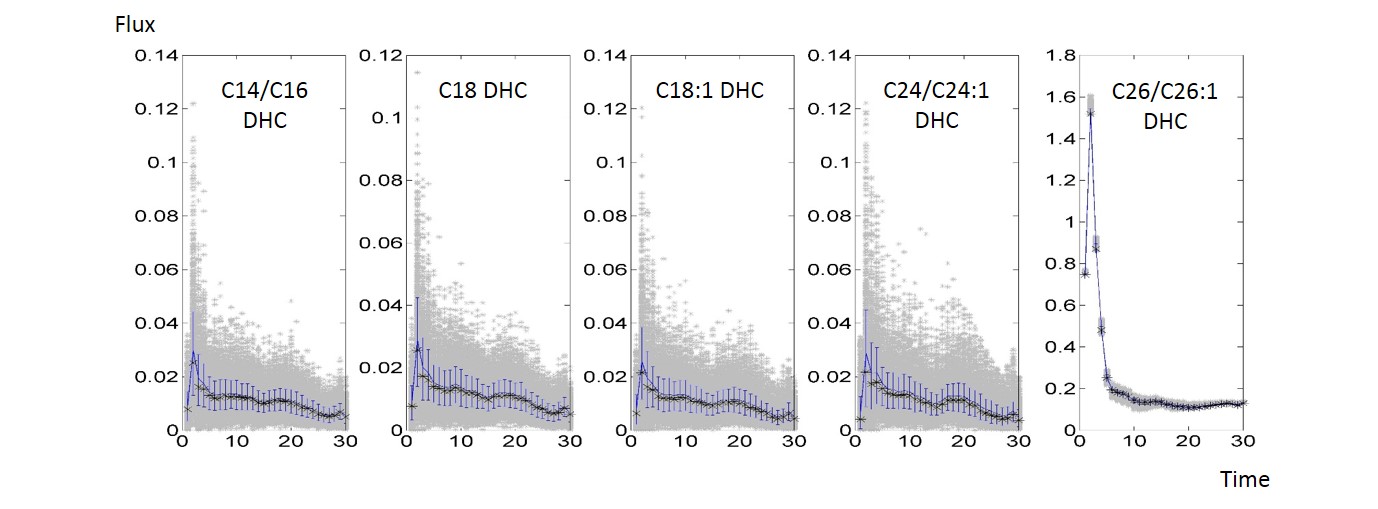
**

**Elongases 1, 2, 3, Remodelase, and Desaturase**

**
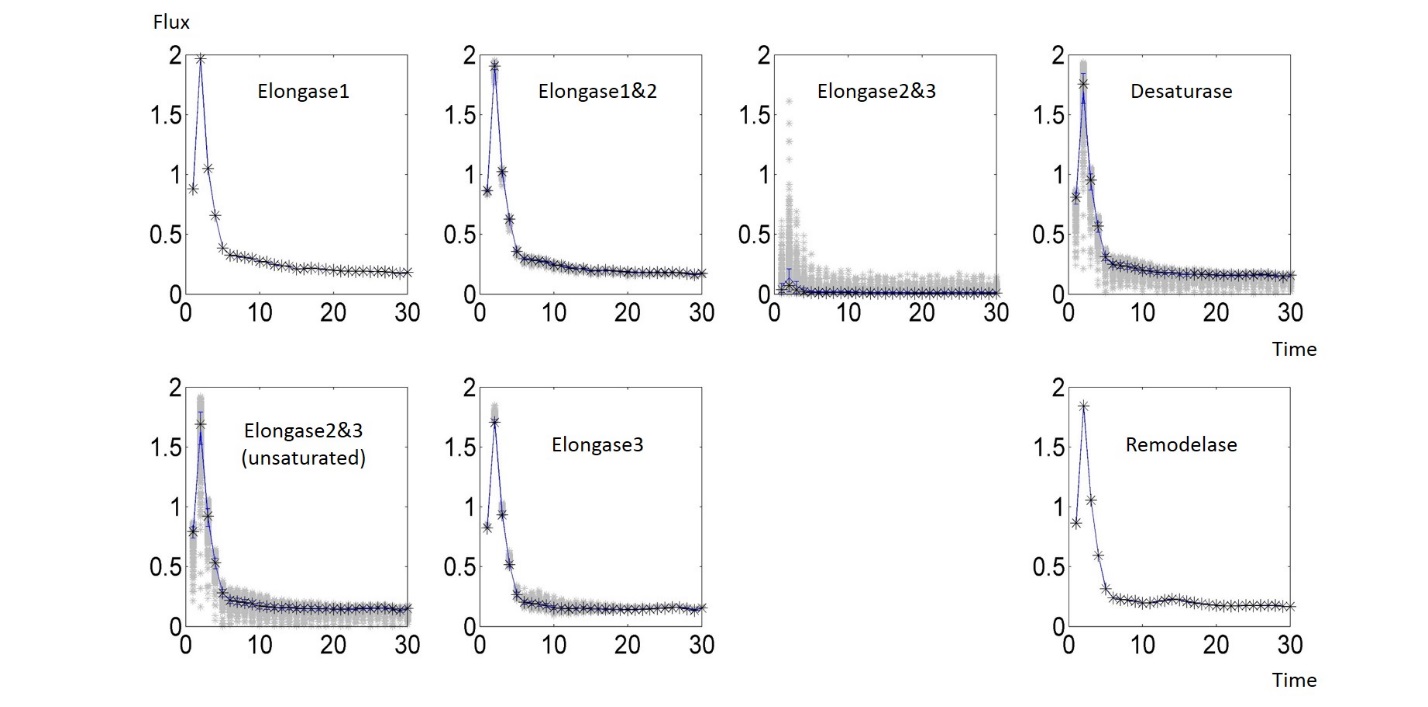
**

**Figure S1: Distributions of fluxes not shown in the main text.** Compare with Figs. 4-9 in the Text.
