# Supplementary material for: Dynamics of the Heat Stress Response of Ceramides with Different Fatty-Acyl Chain Lengths in Baker’s Yeast
Source: PLoS Comput Biol. 2015 Aug 4;11(8):e1004373. doi: 10.1371/journal.pcbi.1004373 (PMC4524633; doi:10.1371/journal.pcbi.1004373)
Supplement: S3 Text — (DOCX) [file pcbi.1004373.s003.docx]

**Supplements**

**Dynamics of the Heat Stress Response of Ceramides with Different Fatty-Acyl Chain Lengths in Baker’s Yeast**

**Po-Wei Chen, Luis L. Fonseca, Yusuf A. Hannun, Eberhard O. Voit**

**S3 Text: Magnitudes of enzyme activities**

It was already mentioned in the main text that the actual enzyme amounts and rate constants are not known. As it is typical, we assume that enzyme activities enter a flux representation in a linear manner. With this assumption we obtain coarse estimates of the product of the rate constant and the enzyme activity. This product corresponds to a *V_max_* value, which by definition consists of the product of *k_cat_* and the total enzyme concentration. Coarse estimates of the ceramide synthase activities are presented in the main text; all other enzyme activities are shown here. Gray dots (which merge into a grey region in each plot) show the individual simulation results; blue lines indicate the means; vertical blue bars correspond to the 20^th^ and 80^th^ percentiles; black asterisks are medians. It is clear that the means and medians are quite similar.

**Ceramide Synthase**


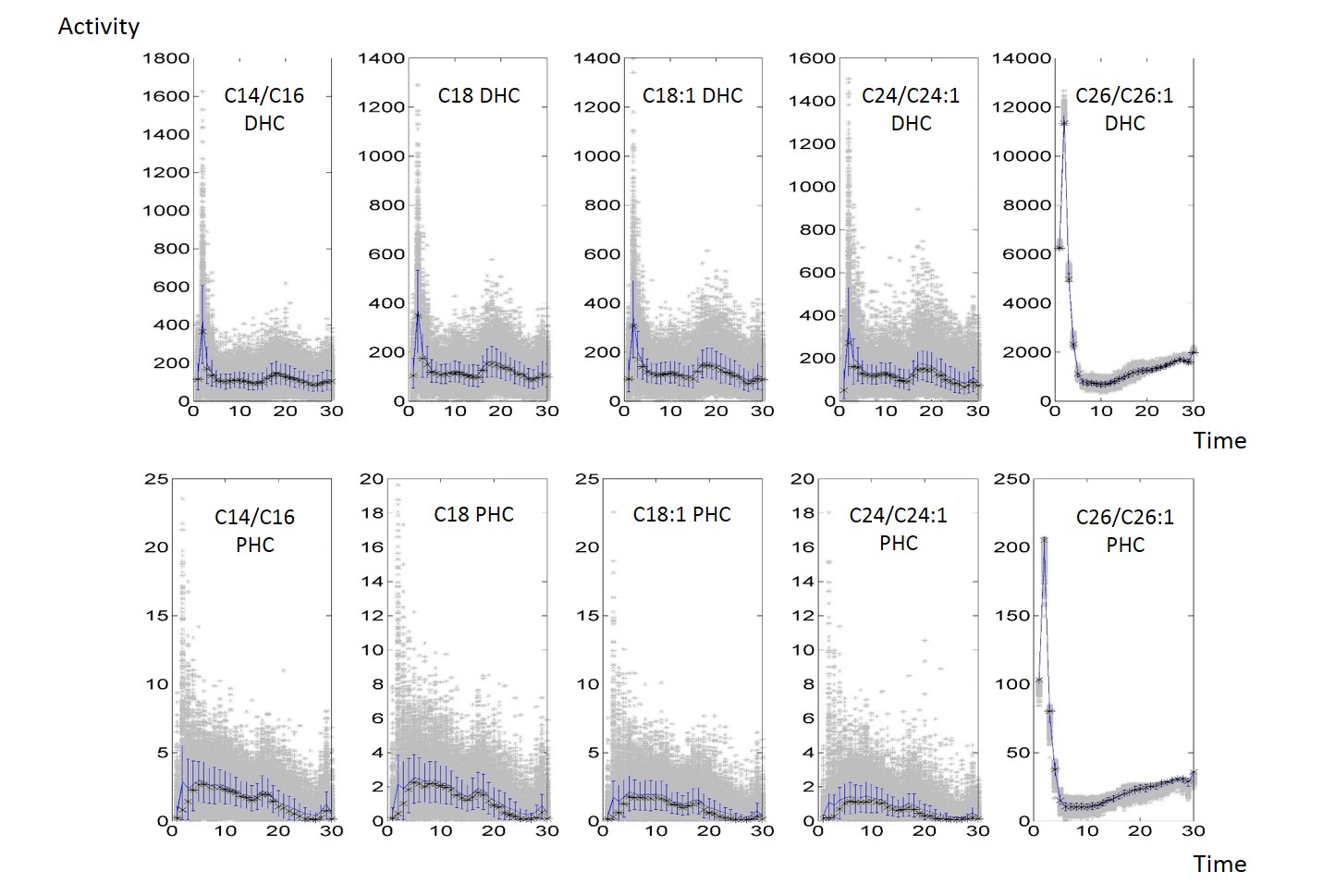


**Dihydroceramidase & Phytoceramidase**

**
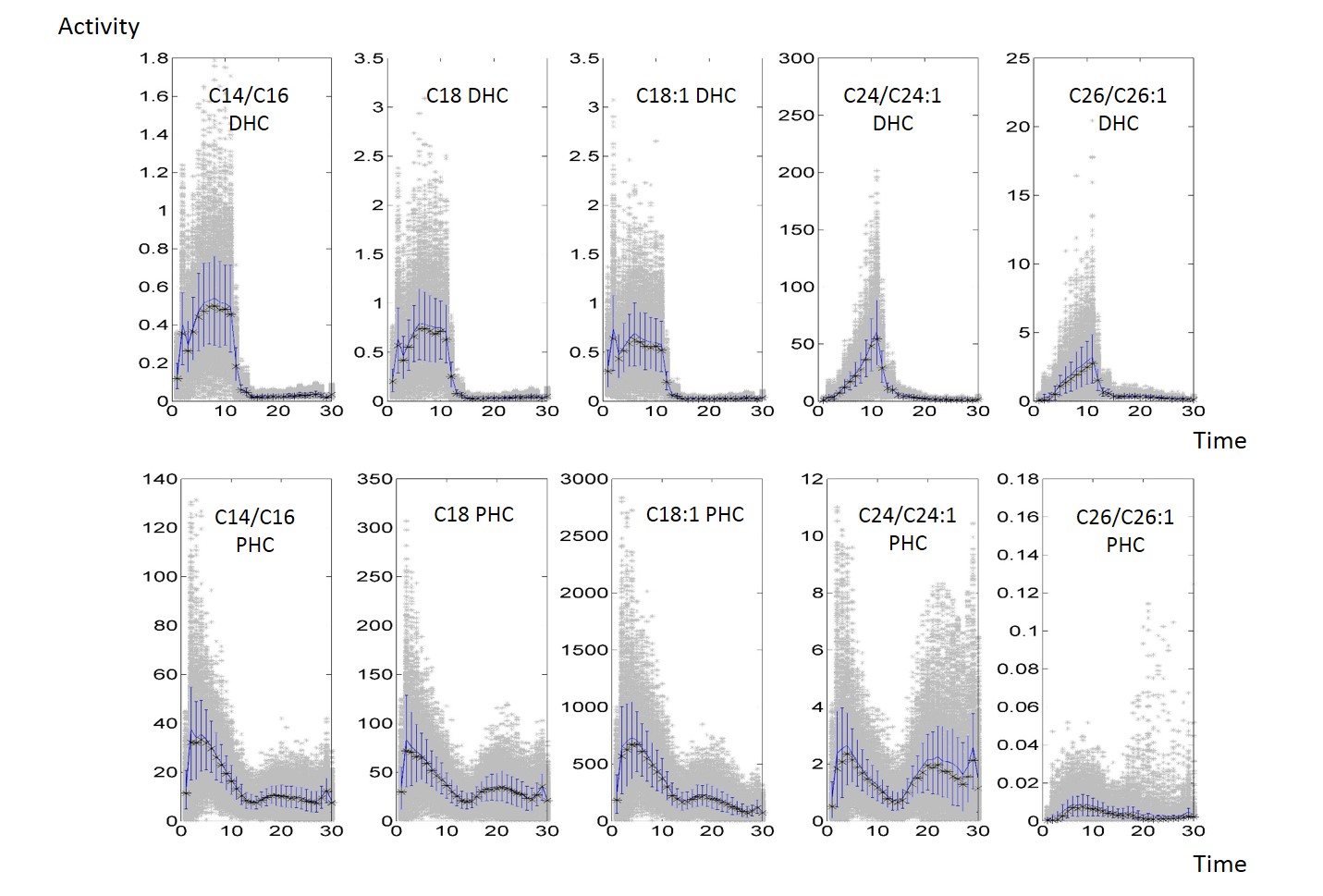
**

**IPC synthase**

**
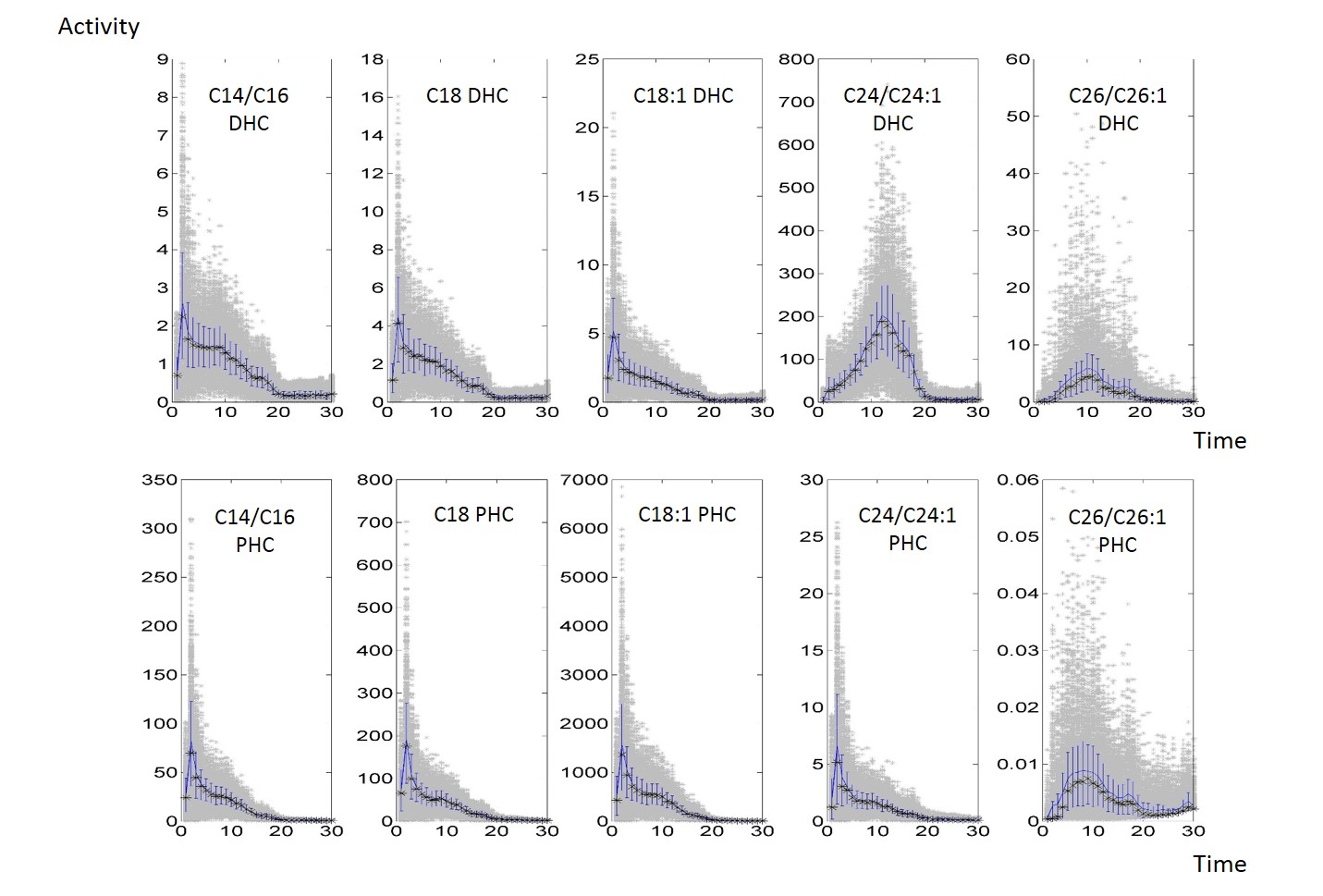
**

**IPCase**

**
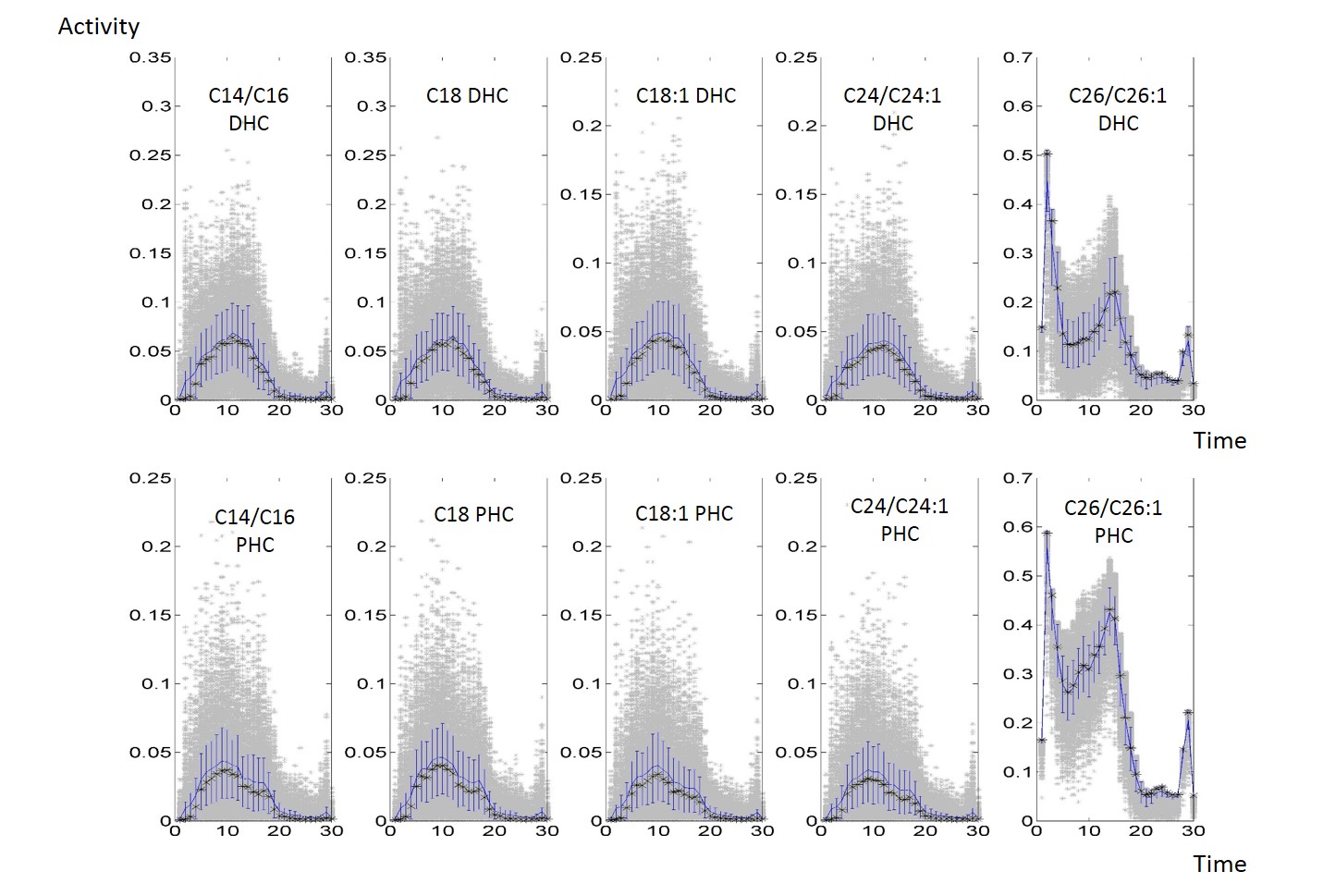
**

**Hydroxylase**

**
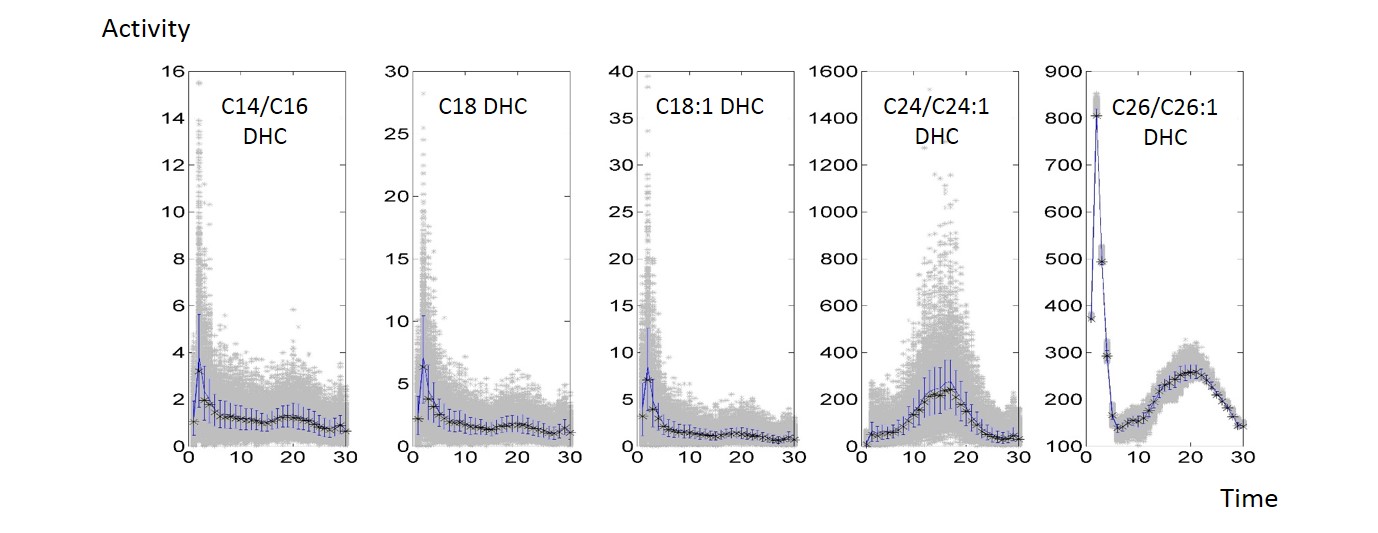
**

**Elongases 1, 2, 3, Remodelase, and Desaturase**

**
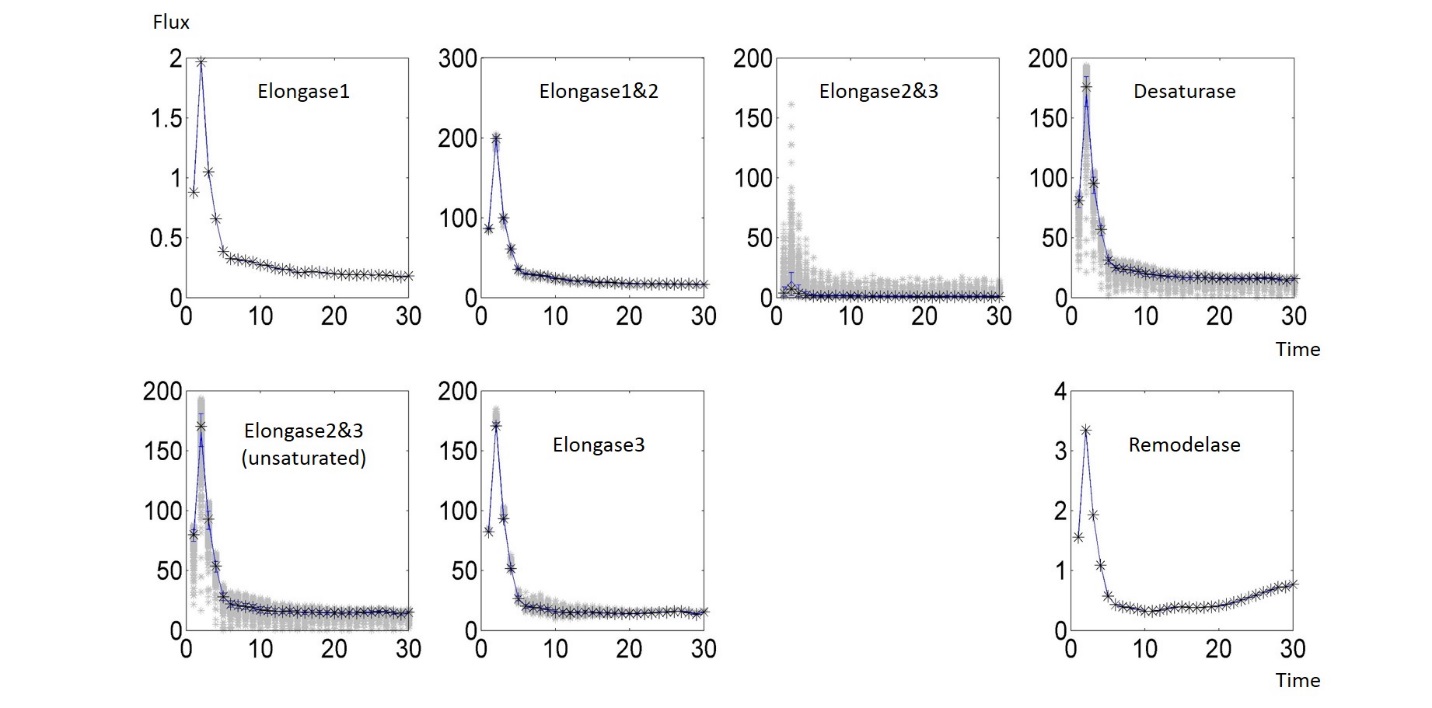
**

**Figure S2: Simulation results for enzyme activities over time, as well as means (blue), medians (black asterisks), and 20^th^ and 80^th^ percentiles (blue bars).** Compare with Fig. 11 in the Text.
