# Supplementary material for: Dynamics of the Heat Stress Response of Ceramides with Different Fatty-Acyl Chain Lengths in Baker’s Yeast
Source: PLoS Comput Biol. 2015 Aug 4;11(8):e1004373. doi: 10.1371/journal.pcbi.1004373 (PMC4524633; doi:10.1371/journal.pcbi.1004373)
Supplement: S4 Text — (DOCX) [file pcbi.1004373.s004.docx]

**Supplements**

**Dynamics of the Heat Stress Response of Ceramides with Different Fatty-Acyl Chain Lengths in Baker’s Yeast**

**Po-Wei Chen, Luis L. Fonseca, Yusuf A. Hannun, Eberhard O. Voit**

**S4 Text: Histograms**

All histograms of each flux, from time point 0 to 30, were examined in order to ensure that the estimated fluxes were unimodal and well constrained in terms of a small variance. The main text showed the histograms of fluxes from DHS to C16 DHC and PHS to C16 PHC at the 0^th^, 5^th^, 10^th^, 15^th^, 20^th^, 25^th^ and 30^th^ minutes of heat stress. The histograms of all 53 fluxes are given here.

**Ceramide synthase**

**
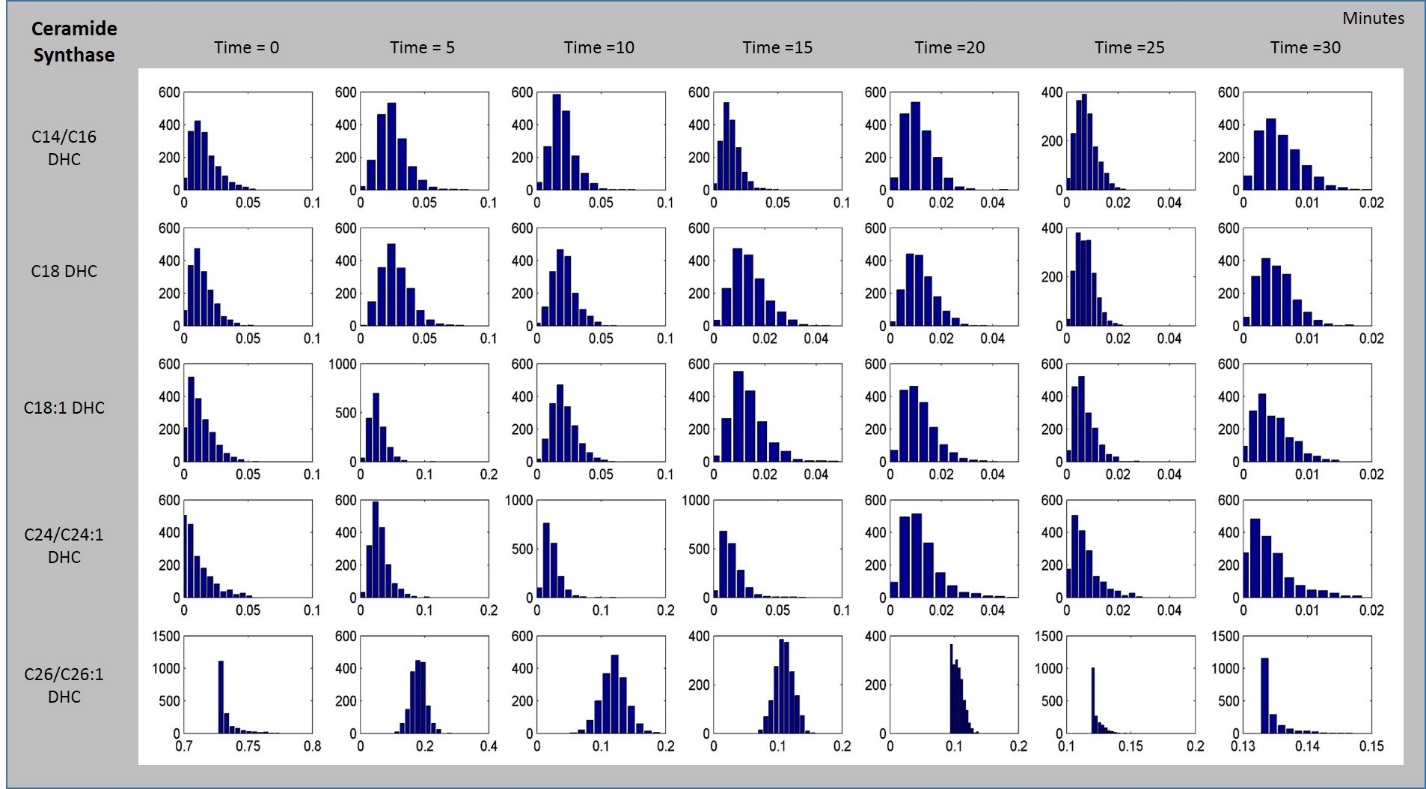
**

**
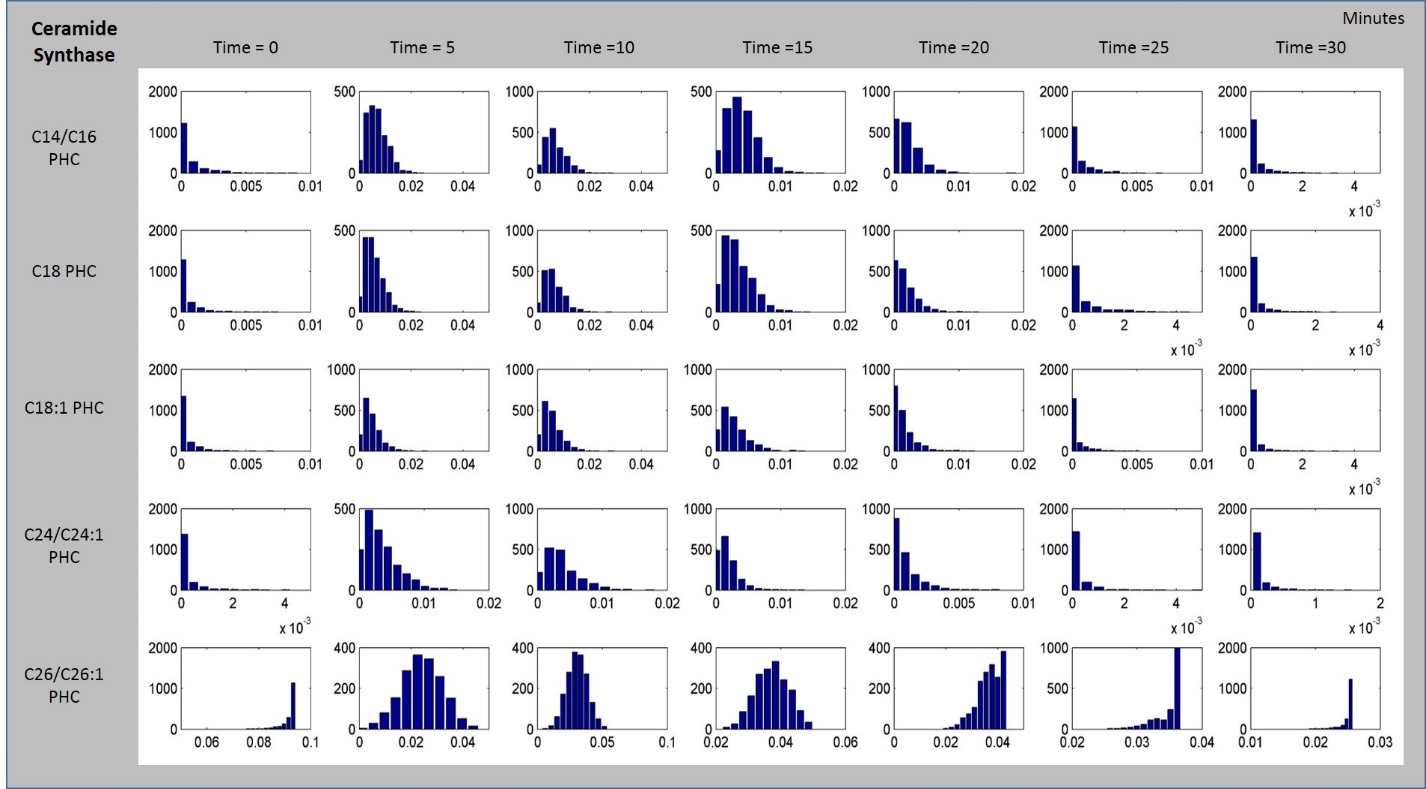
**

**Dihydroceramidase & Phytoceramidase**

**
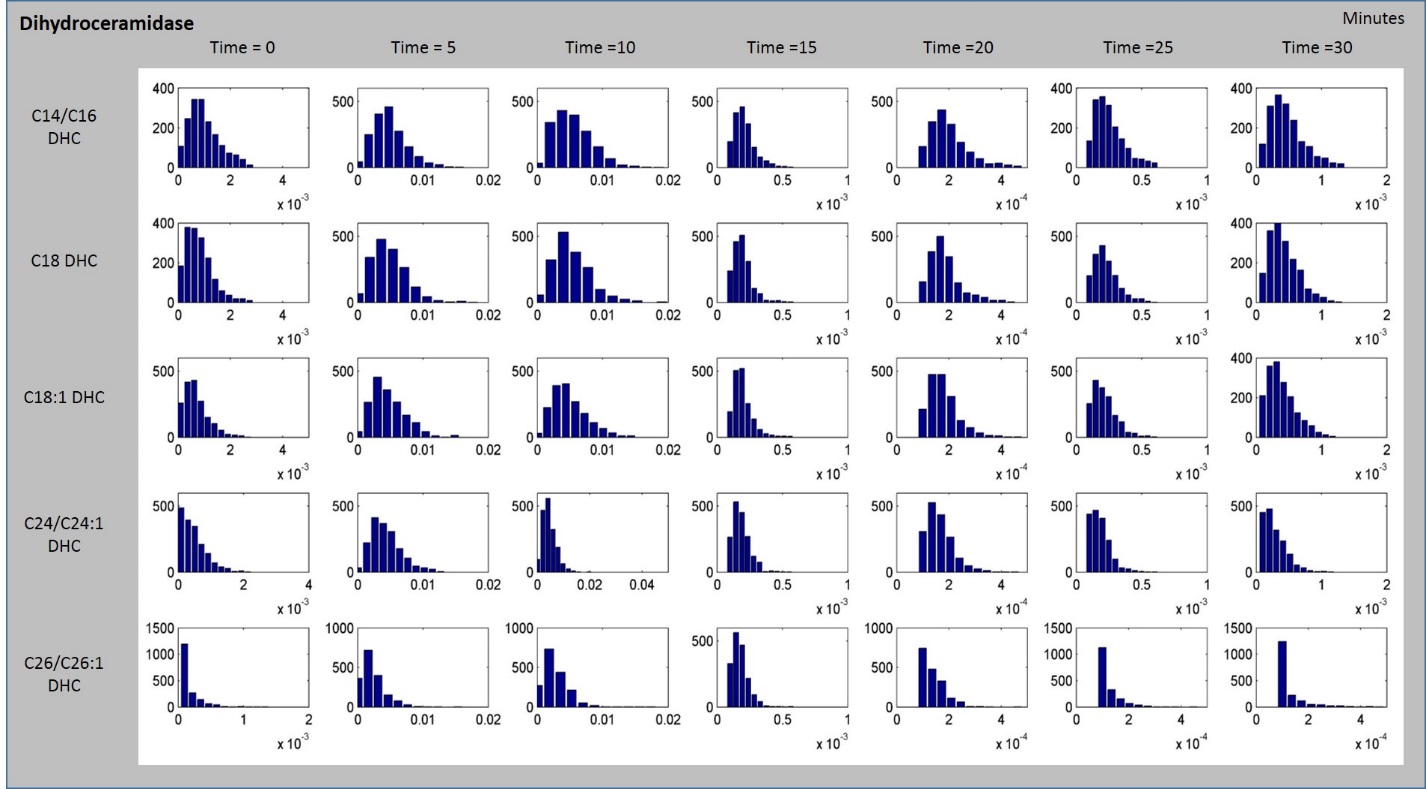
**

**
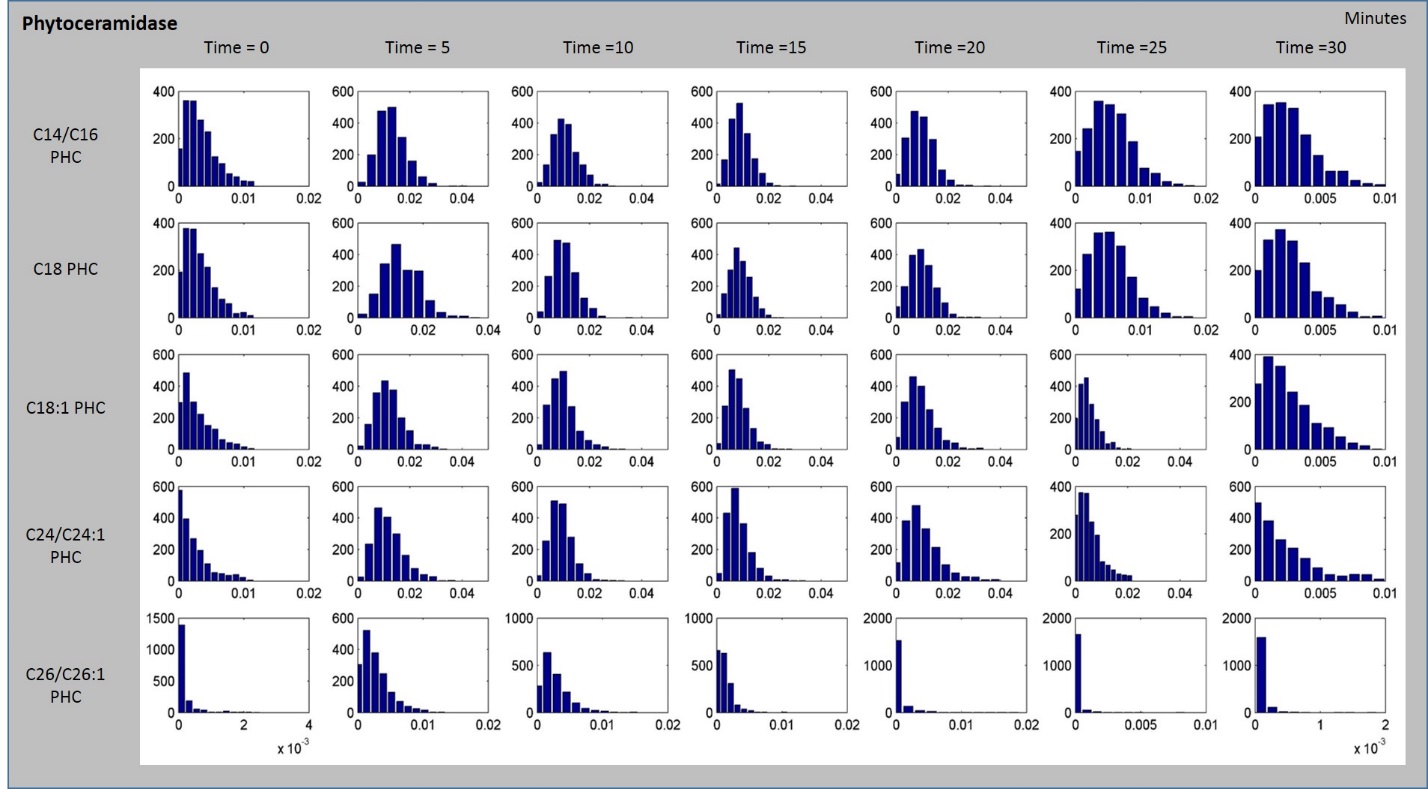
**

**IPC synthase**

**
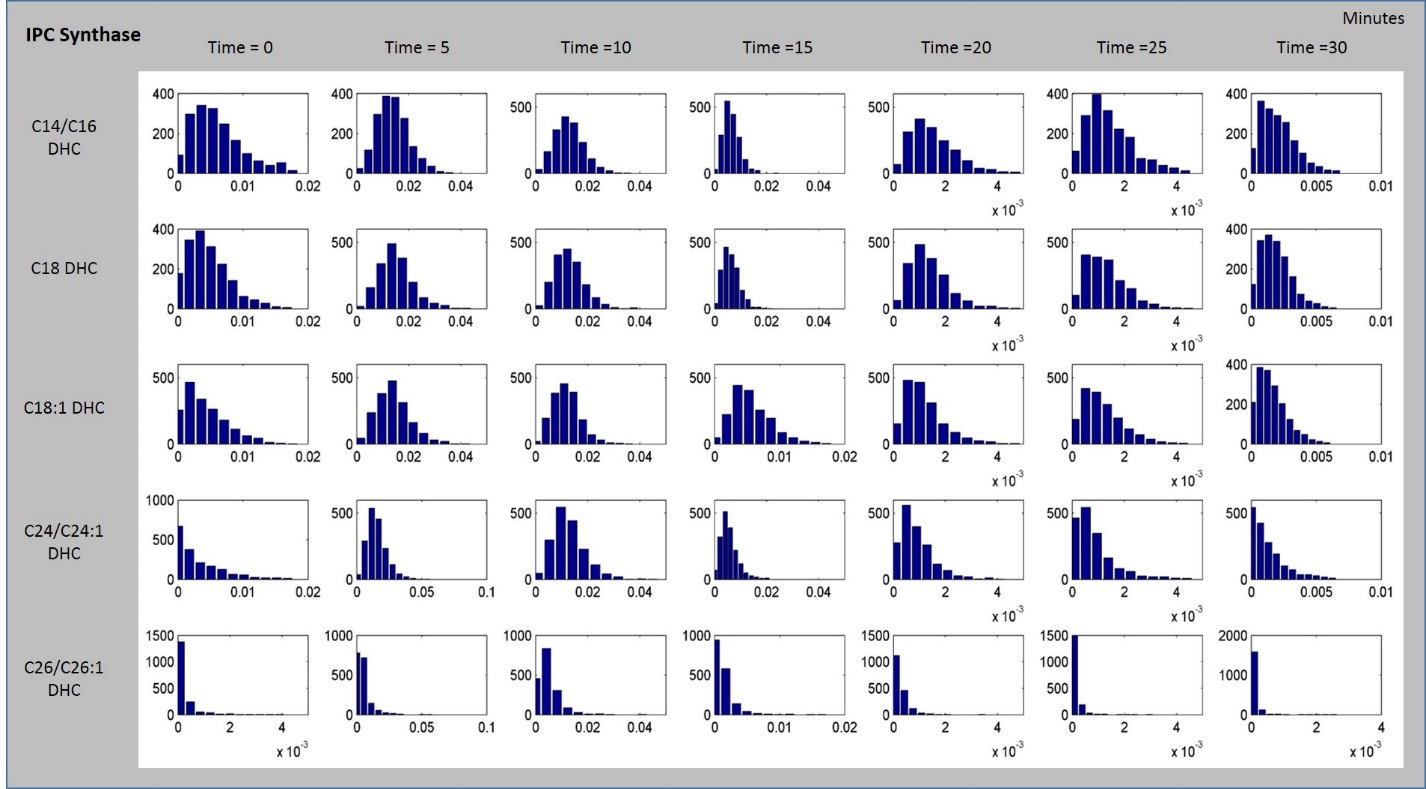
**

**
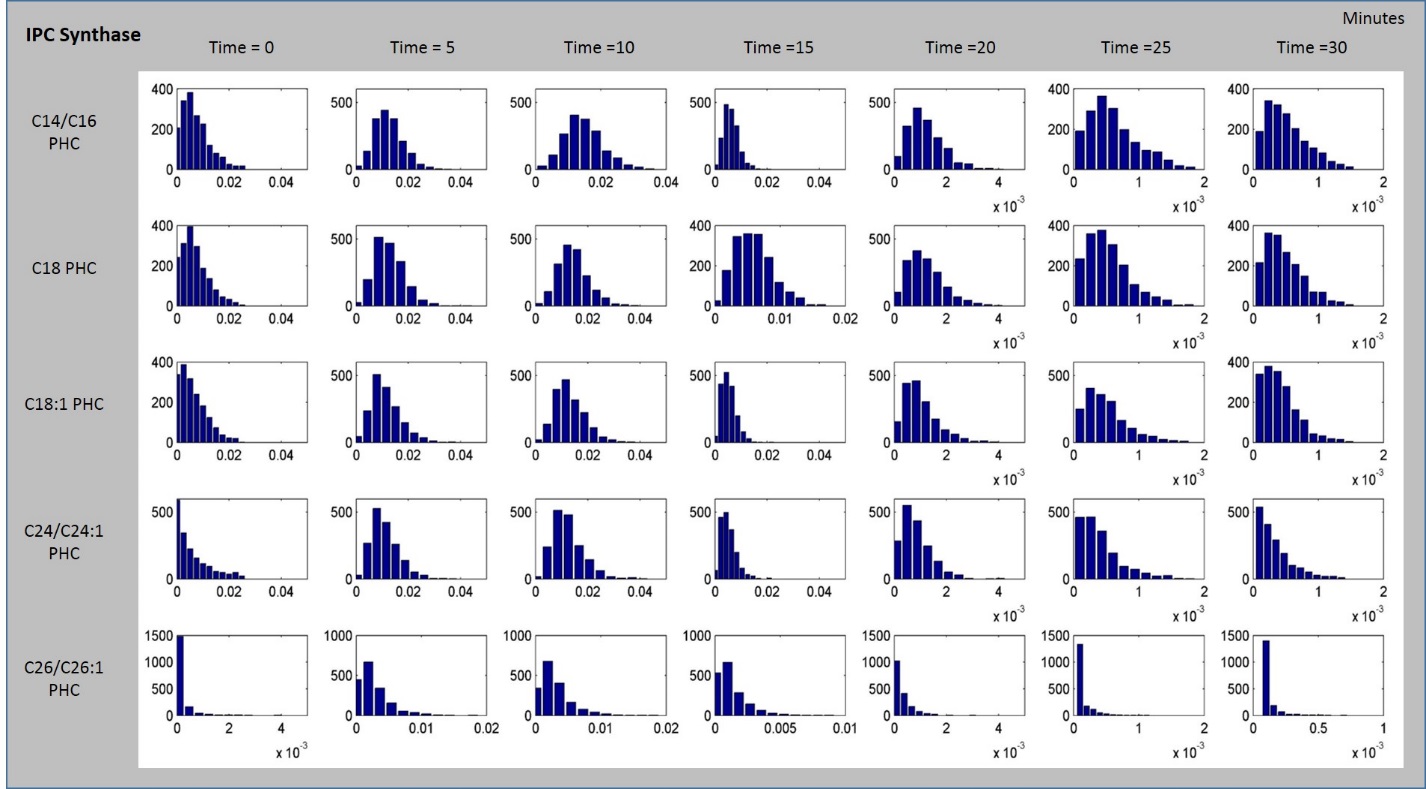
**

**IPCase (Isc1)**

**
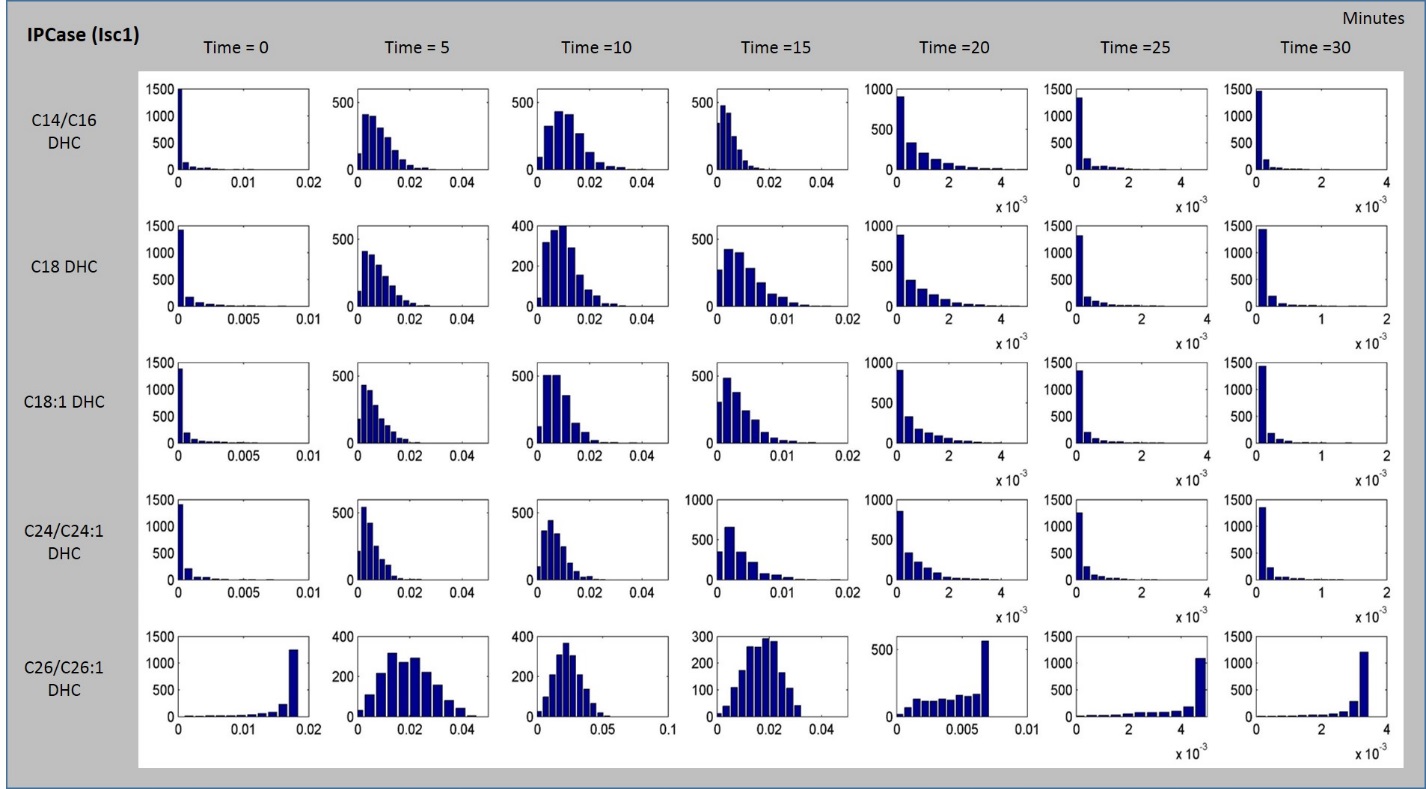
**

**
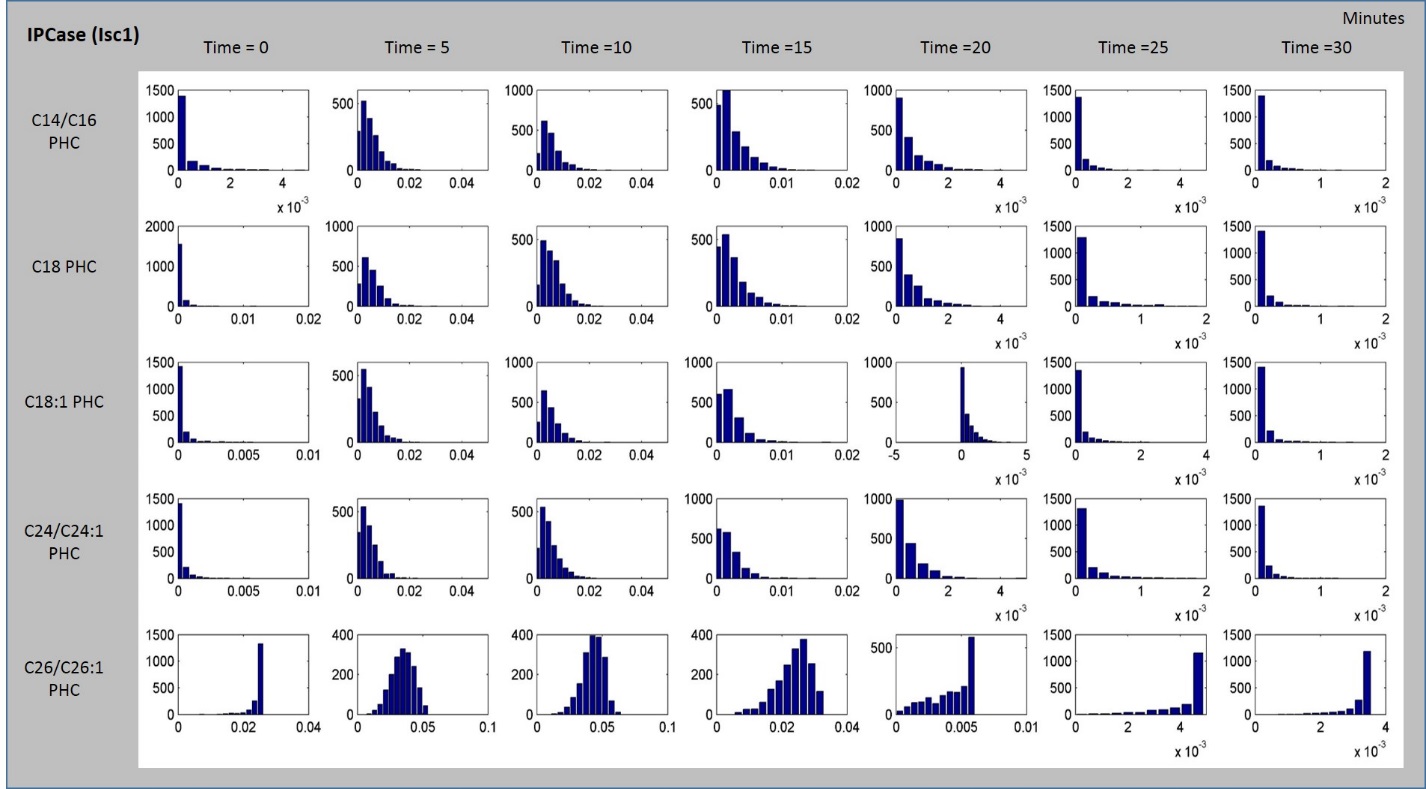
**

**DHC Hydroxylase**

**
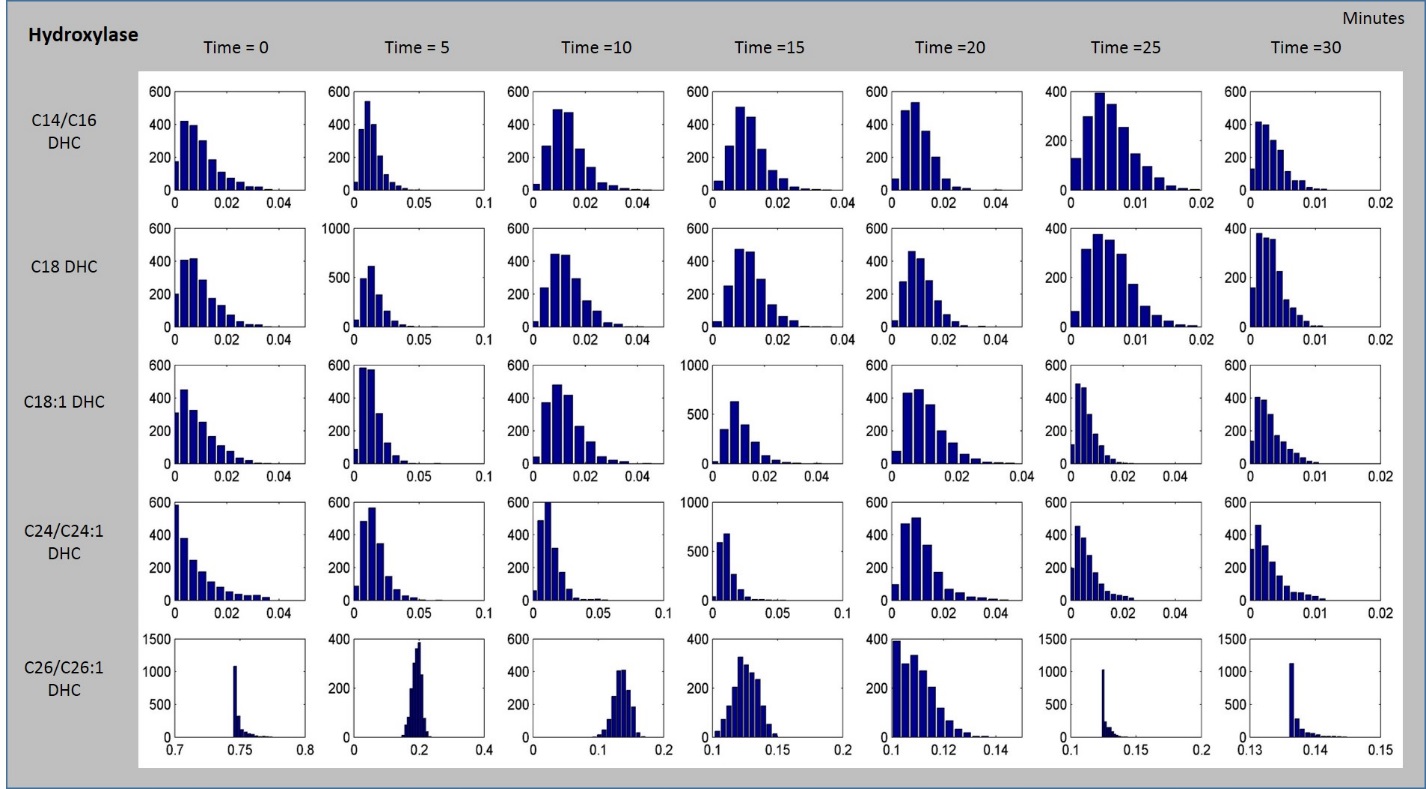
**

**Elongases 1, 2, 3, Remodelase, and Desaturase**

**
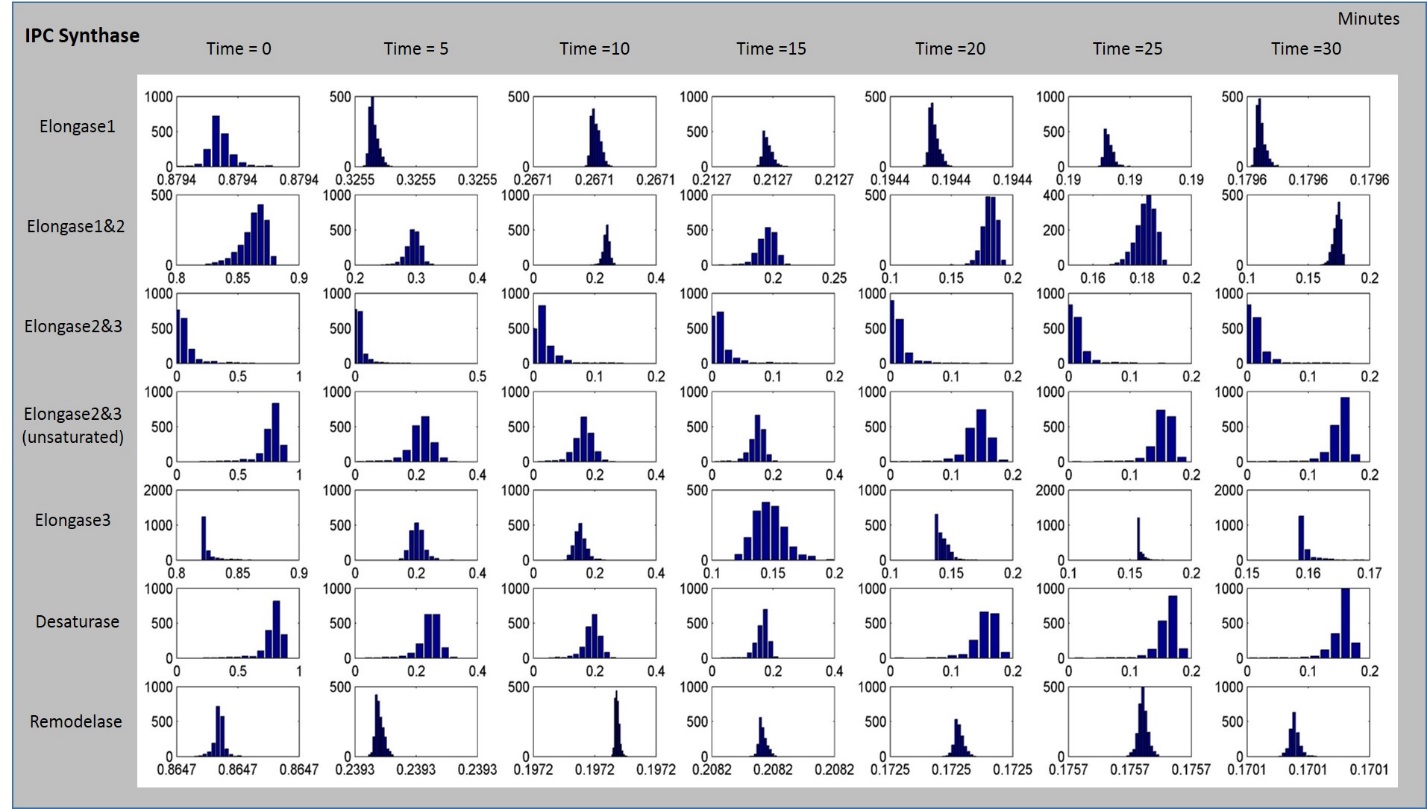
**

**Figure S3: Histograms of fluxes at all measured time points.** Compare with Fig. 12 in the Text.
