# Supplementary material for: Dynamics of the Heat Stress Response of Ceramides with Different Fatty-Acyl Chain Lengths in Baker’s Yeast
Source: PLoS Comput Biol. 2015 Aug 4;11(8):e1004373. doi: 10.1371/journal.pcbi.1004373 (PMC4524633; doi:10.1371/journal.pcbi.1004373)
Supplement: S5 Text — (DOCX) [file pcbi.1004373.s005.docx]

**Supplements**

**Dynamics of the Heat Stress Response of Ceramides with Different Fatty-Acyl Chain Lengths in Baker’s Yeast**

**Po-Wei Chen, Luis L. Fonseca, Yusuf A. Hannun, Eberhard O. Voit**

**S5 Text: Impact of data variability on estimated distributions of dynamic fluxes**

Results shown in the main text are calculated based on interpolation of mean of the duplicate raw data (Figure 10). In order to assess the impact of data variability, we performed the following step-wise analysis:

1. Adopt the original, smoothed and interpolated mean data as the baseline concentration.
2. Sample a uniformly distributed random variable from the range [1/1.5, 1.5].
3. Perturb the original dataset from time point 0 to 30 by the drawn random number and repeat for 1000 simulations.
4. For each set of randomly perturbed data, redo the simulation in the “Methods” Section to obtain dynamic fluxes.
5. Compare the fluxes based on perturbed data with the originally estimated fluxes.

The perturbed data are presented in Figure S4.1, and the perturbed flux distributions are shown in Figure S4.2. Gray dots, blue lines, blue bars and black asterisks represent single simulations, mean values, 20^th^ and 80^th^ percentiles, and median values, respectively. The flux distributions obtained from perturbed data are similar to the originally estimated fluxes (used in the main text). Increased variances are observed, but they do not influence the further estimation of enzyme activities.

**Figure S4.1: Perturbed dataset based on 1.5 fold variation of the interpolated mean data.**

**Ceramide Synthase**

**
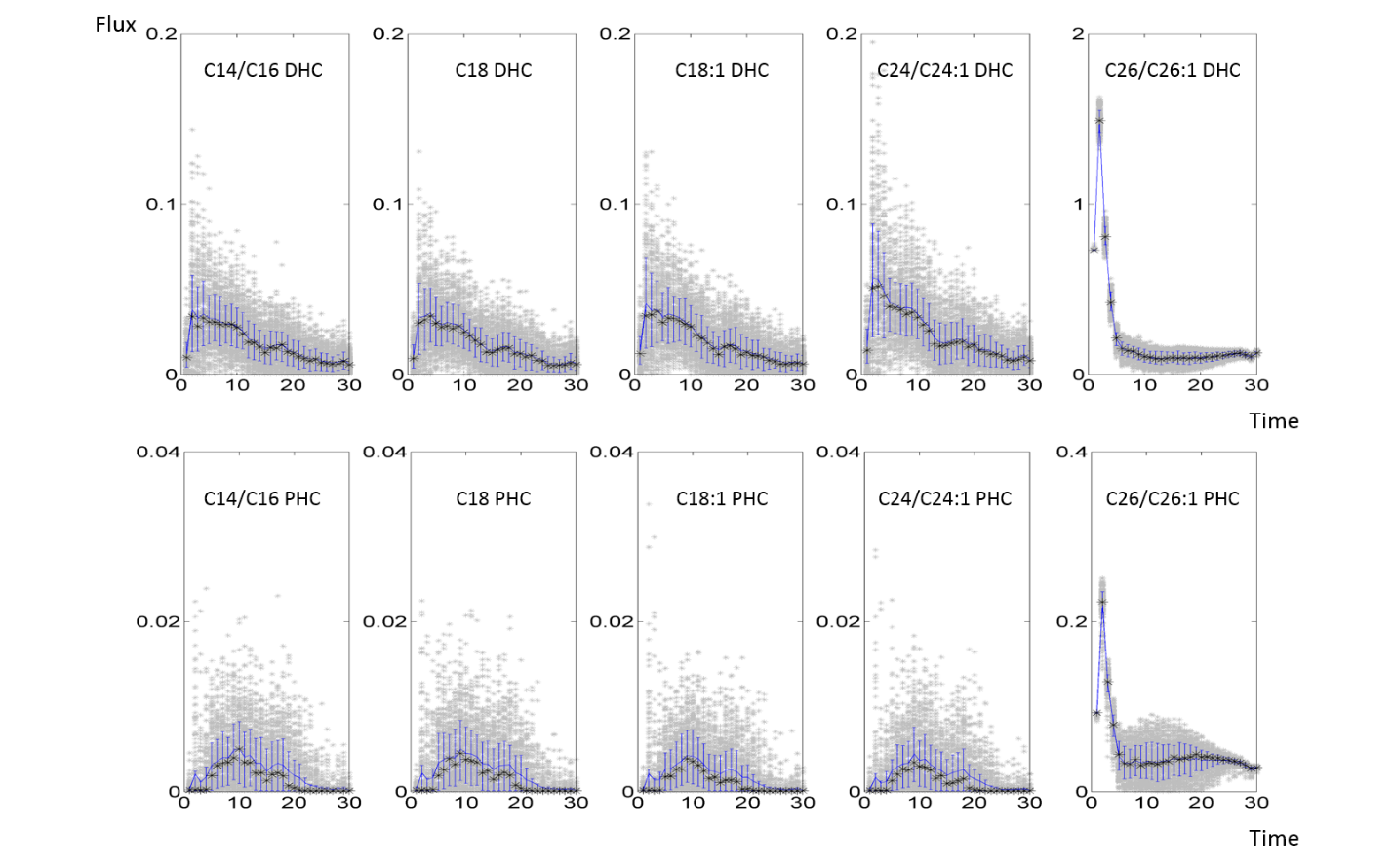
**

**Dihydroceramidase and Phytoceramidase**

**
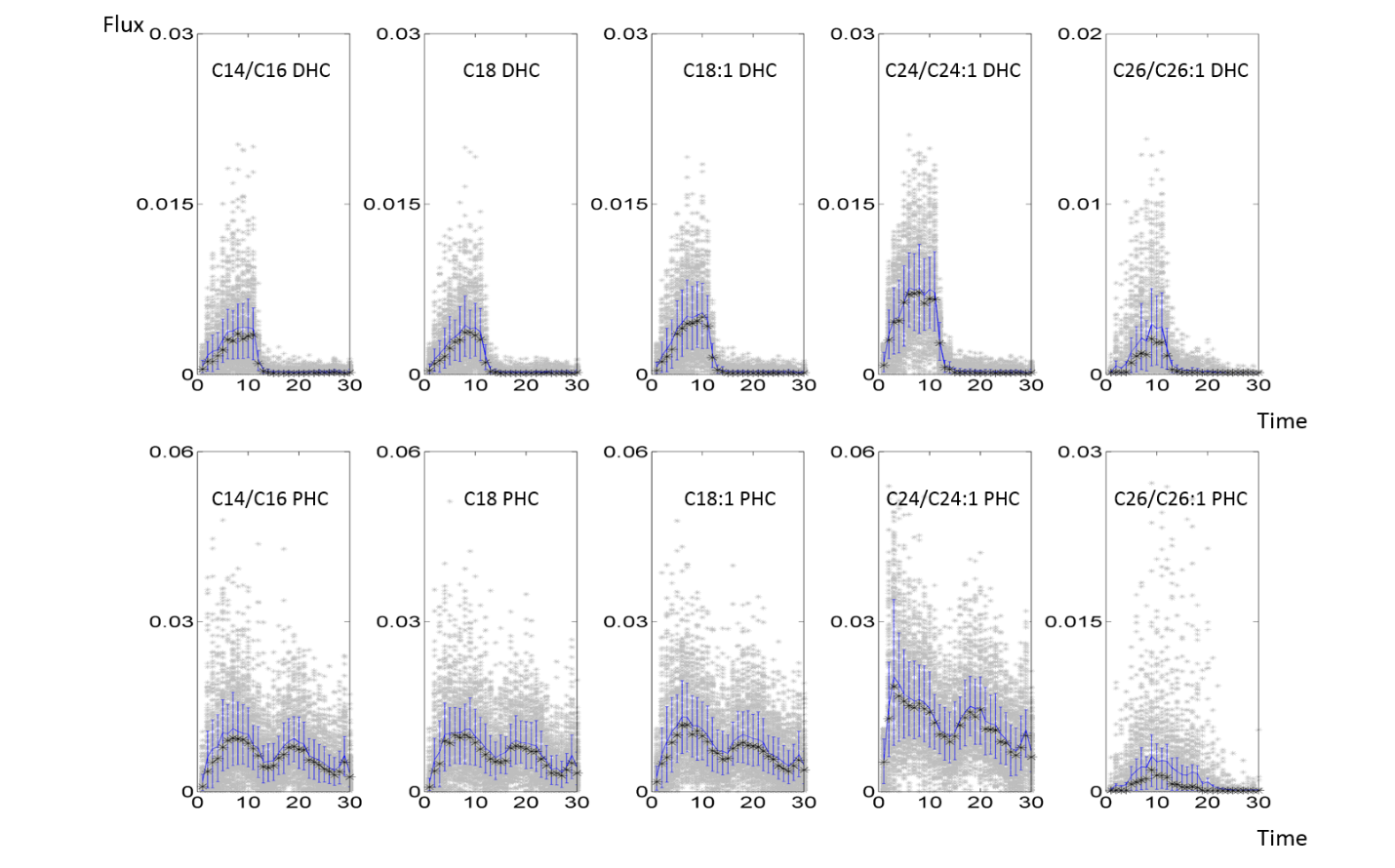
**

**IPC Synthase**

**
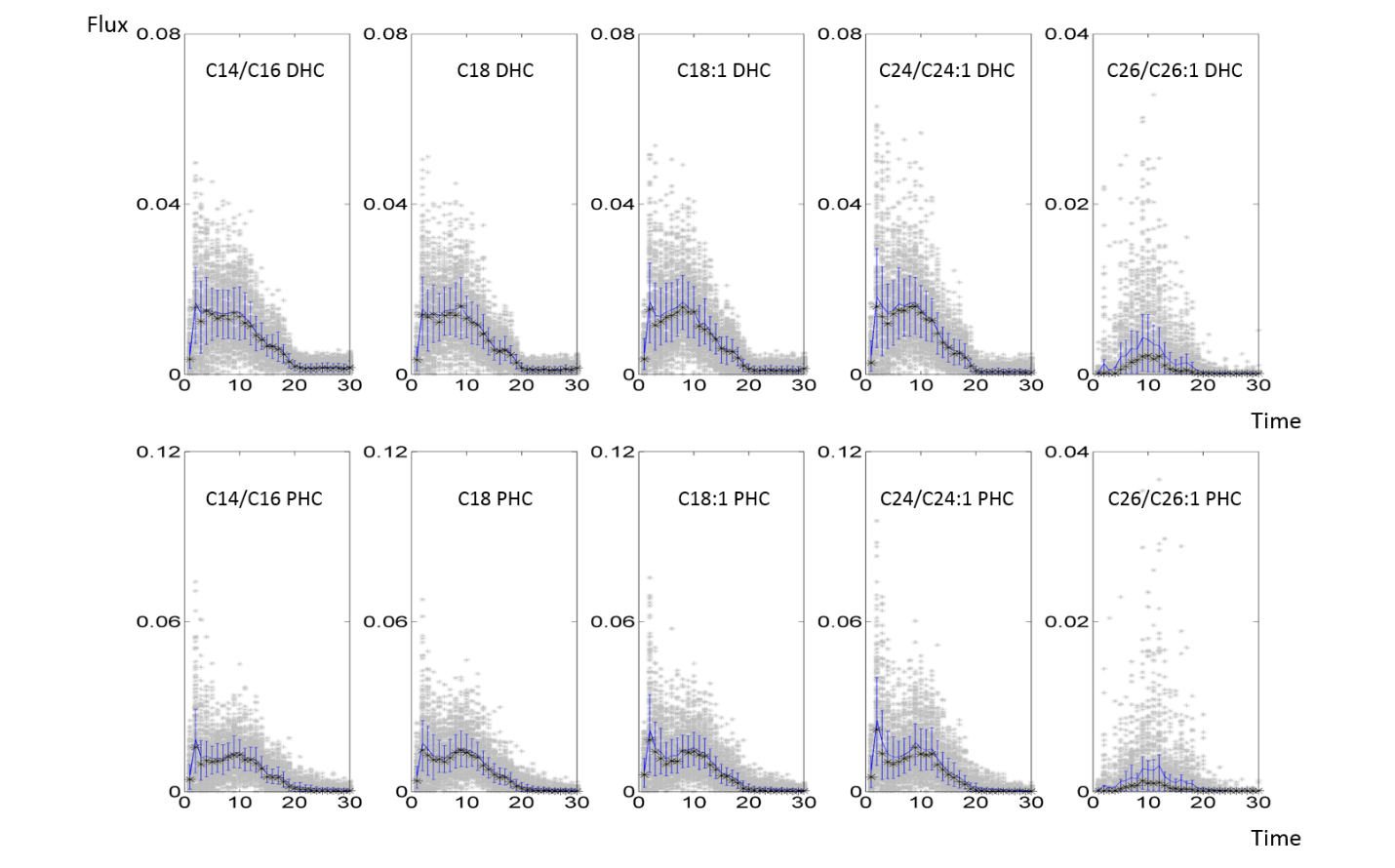
**

**IPCase**

**
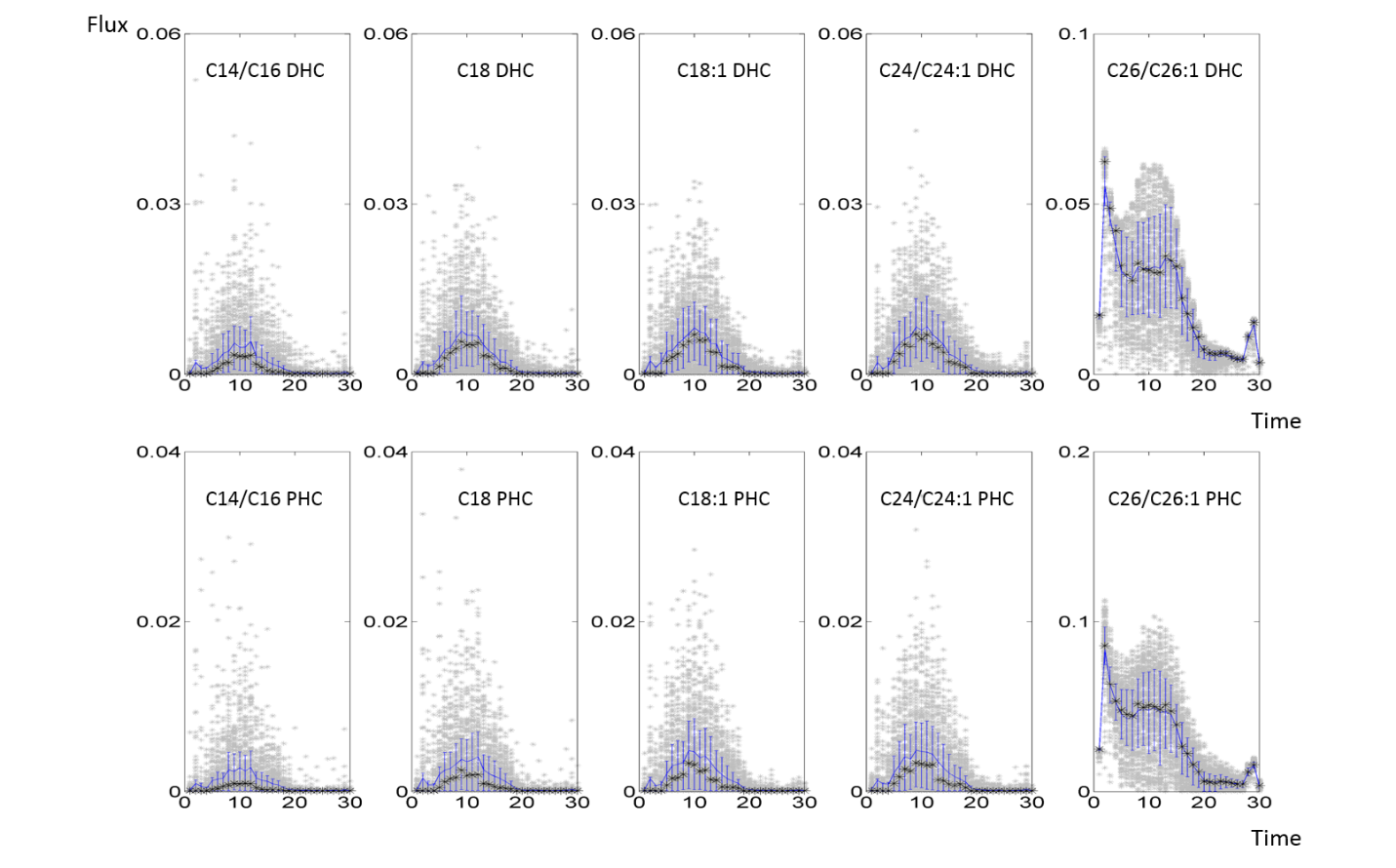
**

**Hydroxylase**

**
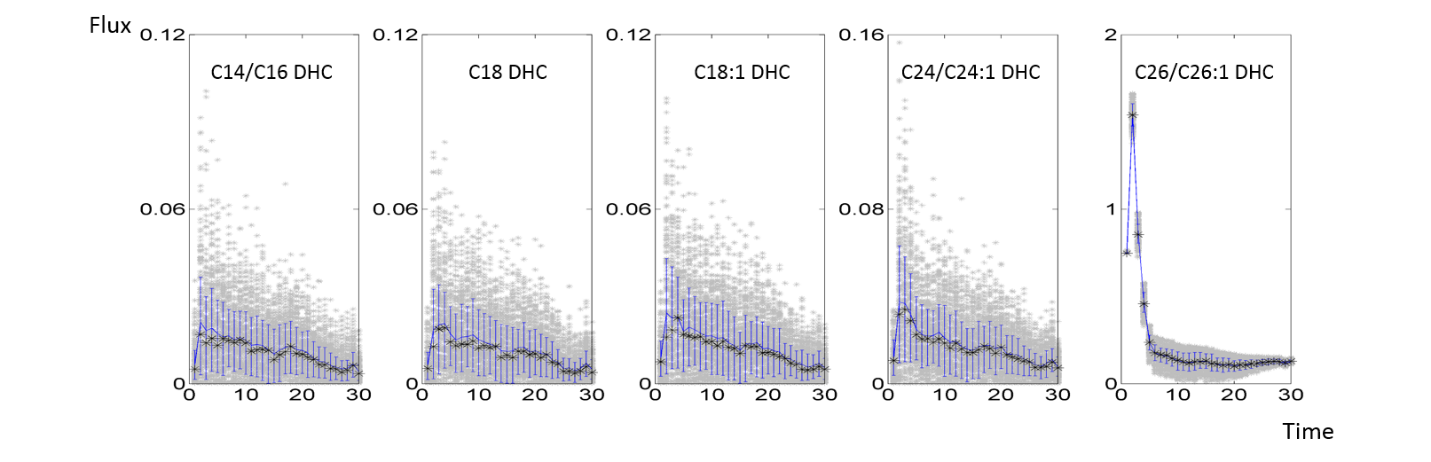
**

**Elongases 1, 2, 3, Remodelase, and Desaturase**

**
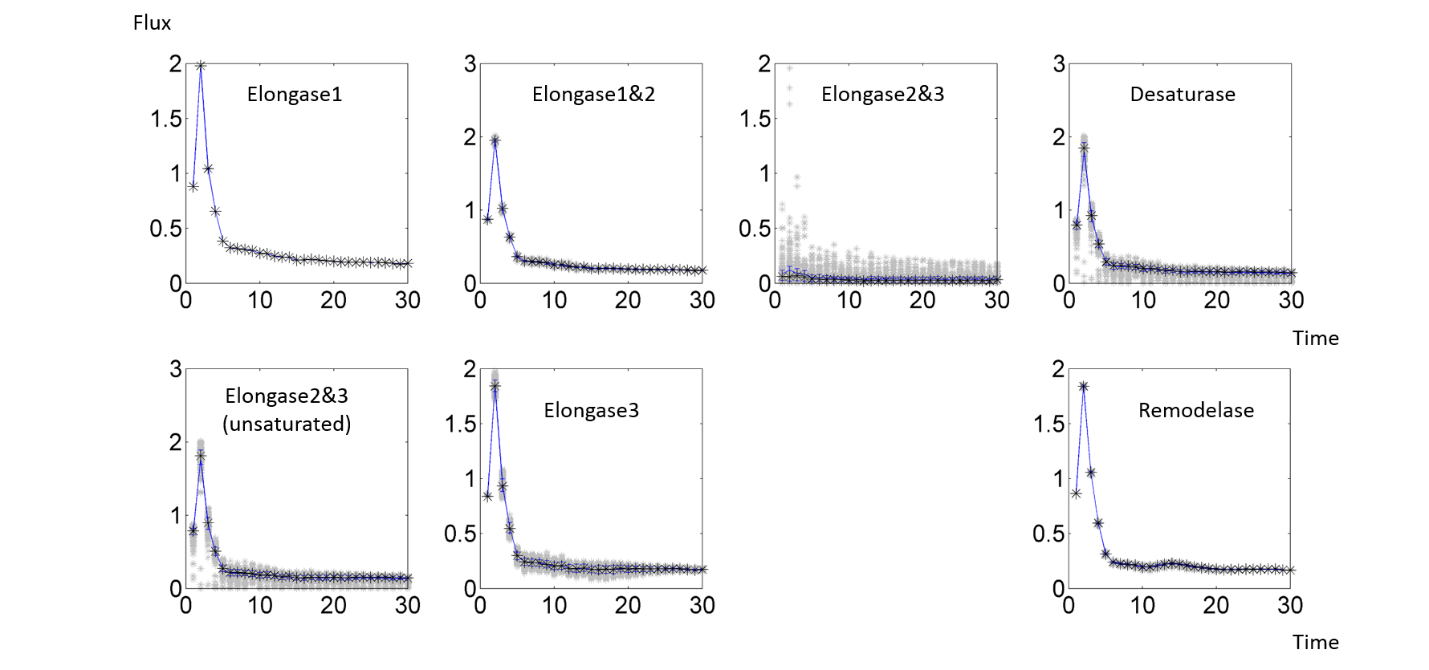
**

**Figure S4.2: Flux distributions calculated from perturbed data set.**
