# Supplementary material for: Dynamics of the Heat Stress Response of Ceramides with Different Fatty-Acyl Chain Lengths in Baker’s Yeast
Source: PLoS Comput Biol. 2015 Aug 4;11(8):e1004373. doi: 10.1371/journal.pcbi.1004373 (PMC4524633; doi:10.1371/journal.pcbi.1004373)
Supplement: S6 Text — (DOCX) [file pcbi.1004373.s006.docx]

**Supplements**

**Dynamics of the Heat Stress Response of Ceramides with Different Fatty-Acyl Chain Lengths in Baker’s Yeast**

**Po-Wei Chen, Luis L. Fonseca, Yusuf A. Hannun, Eberhard O. Voit**

**S6 Text: Justification for using averaged fluxes in the inference of enzyme activities**

Fluxes were estimated 1,000 times at each time point in the interval from 0 to 30 minutes. Averaged fluxes at each time point were adopted as an indication of the dominant trend. To justify this approach, we computed the sum of squared errors (SSEs) for each flux estimation and compared it with the SSE of the averaged fluxes. The results (Fig. S5) clearly show that each that is SSE computed from averaged fluxes is located within the range of SSEs computed from 2,000 individual fluxes between the initial steady state and time point 30. This consistency supports our use of averaged fluxes to represent time dependent changes in the model.

**
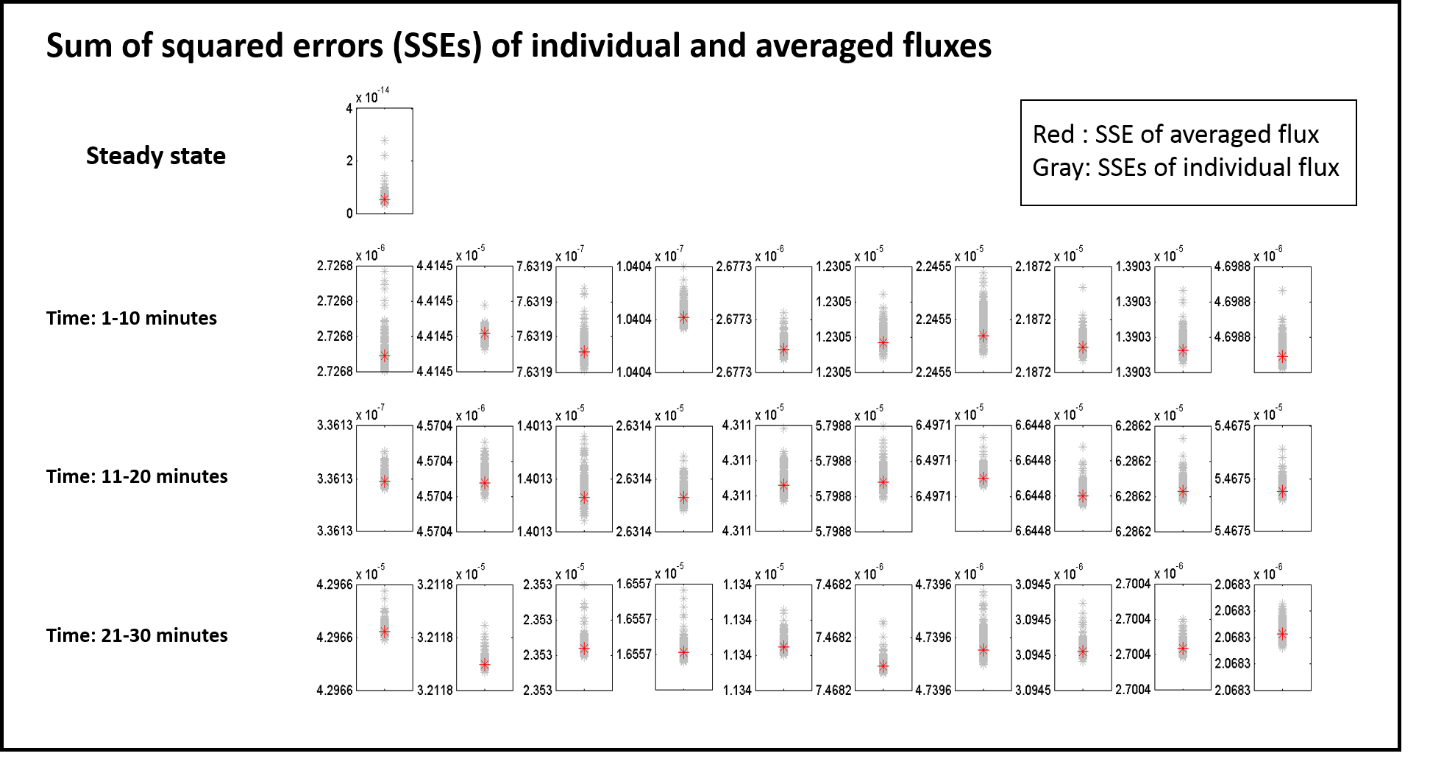
**

**Figure S5: Comparison of sum of squared errors (SSEs) between 2,000 individual fluxes (gray) and the averaged flux (red) at each time point.**
